# The global view of mRNA-related ceRNA cross-talks across cardiovascular diseases

**DOI:** 10.1038/s41598-017-10547-z

**Published:** 2017-08-31

**Authors:** Chao Song, Jian Zhang, Hanping Qi, Chenchen Feng, Yunping Chen, Yonggang Cao, Lina Ba, Bo Ai, Qiuyu Wang, Wei Huang, Chunquan Li, Hongli Sun

**Affiliations:** 1 Department of Pharmacology, Harbin Medical University-Daqing, Daqing, 163319 China; 2 Department of Medical Informatics, Harbin Medical University-Daqing, Daqing, 163319 China

## Abstract

Competing endogenous RNA (ceRNA) have received wide attention because they are a novel way to regulate genes through sharing microRNAs (miRNAs) that are crucial for complex processes in many diseases. However, no systematic analysis of ceRNA mechanism in cardiovascular disease (CVD) is known. To gain insights into the global properties of ceRNAs in multi-CVDs, we constructed the global view of mRNA-related ceRNA cross-talk in eight major CVDs from ~2,800 samples. We found common features that could be used to uncover similarities among different CVDs and highlighted a common core ceRNA network across CVDs. Comparative analysis of hub ceRNAs in each network revealed three types of hubs, which might play key roles in diverse biological processes. Importantly, by combining CVD-related pathway genes with ceRNA-ceRNA interactions, common modules that might exert functions in specific mechanisms were identified. In addition, our study investigated a potential mechanistic linkage between pathway cross-talk and ceRNA cross-talk. In summary, this study uncovered and systematically characterized global properties of mRNA-related ceRNA cross-talks across CVDs, which may provide a new layer for exploring biological mechanisms and shed new light on cardiology.

## Introduction

MicroRNAs (miRNA) play crucial regulatory roles in CVDs^1–3^. MiRNAs are typically 22 nucleotides long and negatively regulate or repress mRNAs or non-coding transcripts by guiding associations between the RNA-induced silencing complex (RISC) and targeting RNAs^4^. Some studies have shown that a group of mRNA transcripts are involved in a ceRNA cross-talk by competing for common miRNA binding sites (also called miRNA response elements, MRE)^5–8^. Some ceRNA cross-talks have been validated from the first discovery of ceRNA in cancer, such as PTEN^9^, FOXO1^10^ and AEG-1^11^. An increasing number of studies have attempted to uncover the mechanism of CVDs in the level of ceRNAs^12,13^. For example, the circRNA HRCR and its ceRNA partners could inhibit cardiac hypertrophy and heart failure by functioning as miRNA sponges of miR-223^14^. Wang *et al*. found that the lncRNA CHRF act as an endogenous sponge of miR-489, down-regulating miR-489 expression and regulating Myd88 expression in cardiac hypertrophy^15^. The lncRNA APF is a sponge of miR-188-3p that decreases degradation of ATG7, which regulates autophagy and myocardial infarction^16^. These studies suggest that ceRNA cross-talk is crucial in miRNA-mediated interactions in CVDs and systematic investigation of the ceRNA cross-talk across CVDs is needed.

CVD, a leading cause of death, encompasses a broad range of conditions from myocardial infarction to congenital heart disease; most CVDs are heritable ^17^. CVD composed more than 10 disease subtypes and investigating the common or specific features across various diseases is important. The rapid development of high-throughput experimental techniques such as microarray and RNA-seq can detect the expression of mRNA transcripts. These techniques have contributed to integrative analysis of molecular ceRNA interactions using gene expression correlations. Studies also provided supports to the research of ceRNA, such as open source data and computational methods. For instance, the open source database starBase provides CLIP-seq supported miRNA-mRNA interactions^18^. Our previous study investigated lncRNA-mRNA ceRNA cross-talks by integrative analysis of gene dysfunction and CLIP-seq-supported miRNA-mRNA/lncRNA interactions in cardiac hypertrophy^19^. In addition, a study proposed a new computational method to identify the sponge interaction by integrating gene co-expression information in breast cancer^20^. Other studies focused on cancer-related ceRNA networks constructed using gene co-expression levels^21,22^. A landscape of mRNA-related ceRNA interactions across 20 cancer types was constructed through integrating TCGA gene expression data^23^. Wang *et al*. identified lncRNA-associated competing triplets across cancers using TCGA data^24^. TCGA stored the data only on multiple cancers. But in the field of CVDs, no database systematically stores gene expression data. Thus global research on CVDs is difficult. Luckily, we obtained gene expression data on CVDs from GEO database, which stored numbers of mRNA-related expression profiles. Compared with the TCGA database, data from GEO is dispersive, thus we can only collect data manually. By performing efficient bioinformatics methods, we obtained gene expression data for investigating the ceRNA cross-talks in multiple CVDs.

In our present study, we performed an integrated analysis of 2,884 samples from eight major CVDs to identify mRNA-related ceRNA cross-talk. First, ceRNA cross-talk networks of each gene expression profile were constructed. We systematically analyzed the topological features and characterized common properties in these ceRNA networks. A common core ceRNA-ceRNA interaction network was identified for various CVDs. After merging the disease associated ceRNA networks, we mapped the CVD-related pathway genes into the networks to identify the common modules that might be crucial for the processes of CVDs using in depth analysis of network structures. In addition, we also found that the ceRNA cross-talks might drive the cross-talks of pathways.

## Results

### MiRNA mediated mRNA-related ceRNA cross-talk in CVDs

**Figure 1:**
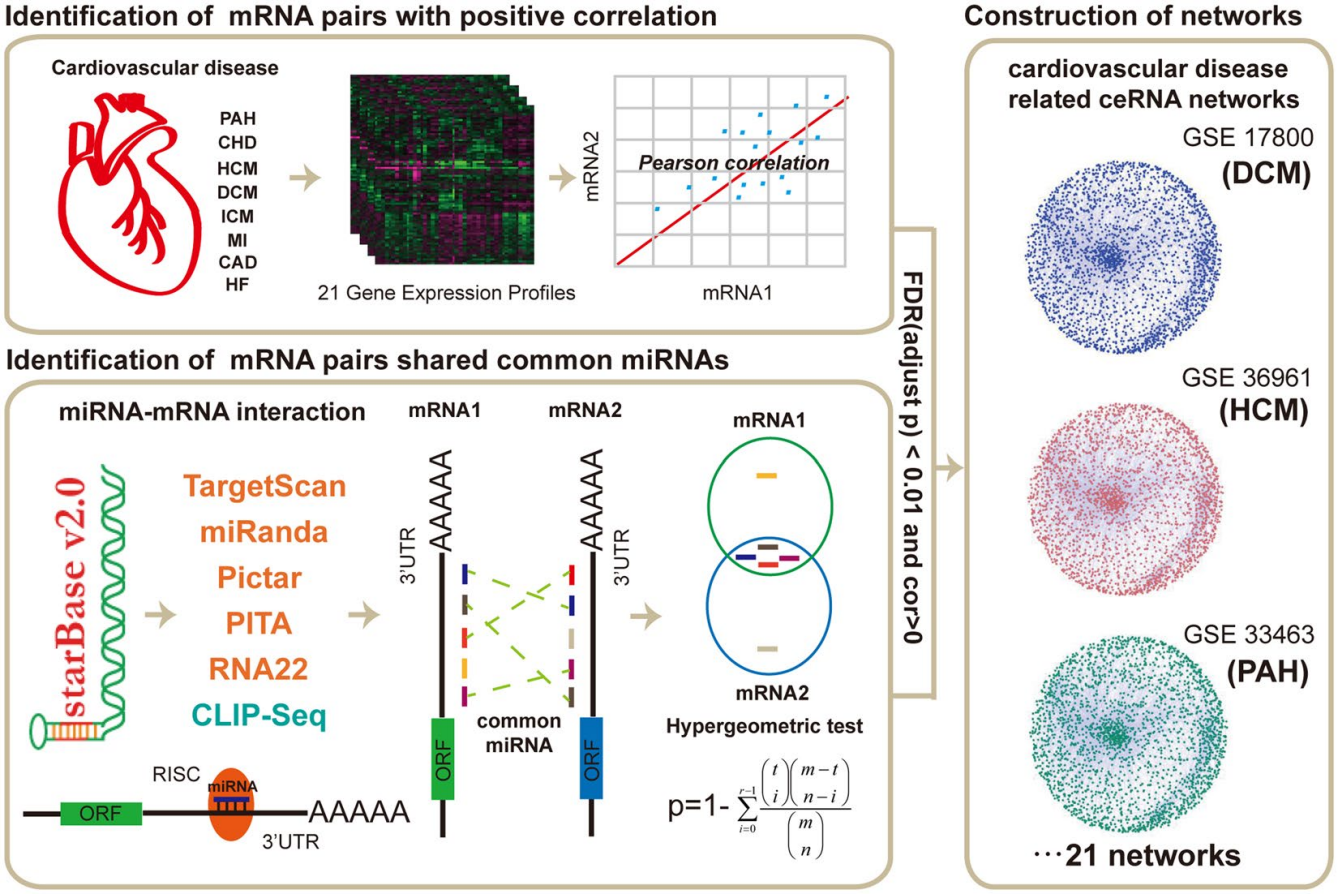
An integrative pipeline for construction of CVD-related ceRNA networks. CLIP-seq-supported miRNA-mRNA interactions were downloaded from starBase. A hypergeometric test was performed to estimate candidate pair mRNA-mRNA relationships with shared common miRNAs. We computed the Pearson correlation coefficients for candidate ceRNA pairs in each of profiles. All the candidate ceRNA pairs with R > 0 and adjusted p-value < 0.01 were identified as ceRNA–ceRNA interactions, and 21 CVD-related ceRNA networks were constructed.

To systematically evaluate the potential role of miRNA-mediated ceRNA cross-talk in multiple CVDs, we performed a two-stage analysis to construct ceRNA cross-talk landscapes for each CVD-associated gene expression profile (Fig. 1, see in Materials and methods). First, 7,846 genes were identified to participate in 511,210 pairwise miRNA mediated RNA-RNA interactions by applying hypergeometric test (FDR < 0.01). In addition, these candidate ceRNA interactions shared more than three common miRNAs. Second, to construct CVD-specific ceRNA interactions, Pearson correlation coefficients were calculated to filter global candidate ceRNA interactions for each CVD-associated gene expression profile based on gene expression values. In second step, we identified ceRNA interactions using cut-off with R > 0 and p-value < 0.01 (We also calculated the FDR values in this step. Results showed that all p-values were significant at the cut-off of FDR < 0.2 (Supplementary Table S1). We merged all ceRNA interactions of each network and removed duplicate interactions. Nearly 74% of all 511,210 candidate ceRNA interactions appeared in the CVD-related ceRNA interactions. In addition, all CVD-related ceRNA interactions implicated with 1.7-69% ceRNA interactions and 15-78% genes (Supplementary Table S1). After the two-stage analysis, 21 CVD-associated ceRNA cross-talk networks were constructed and viewed in Supplementary Fig. S1. All these networks showed high interactivities and were used to investigate the biological mechanism of CVDs.

### Common characters of ceRNA networks

**Figure 2:**
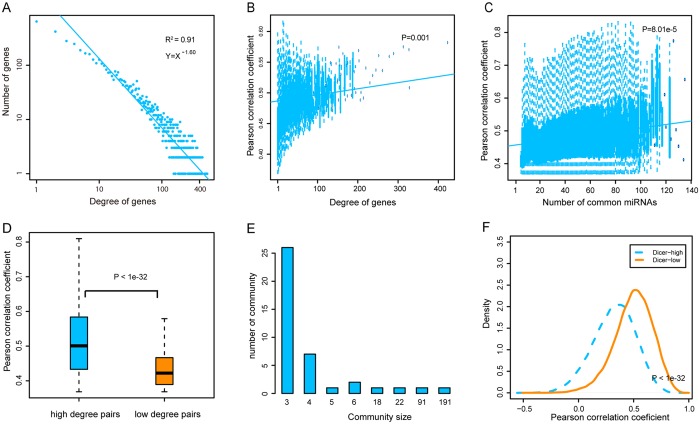
The global topological features of CVD-related ceRNA networks. We selected topological features of a ceRNA network (GSE17800) as a sample and shown in Figure A–F. (**A**) Degree distributions of the ceRNA network. (**B**) CeRNAs with high degrees were strongly co-expressed than others. (**C**) CeRNA pairs shared more miRNAs were strongly co-expressed than others. (**D**) CeRNA pairs with high degrees were strongly co-expressed than others. (**E**) CeRNAs were likely to involve in small communities. (**F**) CeRNA cross-talks preferred co-express in low Dicer expression groups.

Comparison and analysis of the topological characters of the ceRNA cross-talk networks identified common characteristics. First, degree distributions of all networks followed power law distributions (Fig. 2A and Supplementary Fig. S2), indicating that ceRNA networks were scale free. A handful of nodes with high degree in the networks were defined as hubs that linked many nodes; most nodes in networks had few interactions. A gene with more interacting partners or that shared more miRNAs with other genes might exert more functions in biological processes. Also, some studies found that genes tend to compose a module to participate in complex biological regulation because of high connectivity and synergy in diverse disease networks^25,26^. Our analysis of the interactions of ceRNA nodes showed that ceRNAs with higher degree were more highly co-expressed than others in most ceRNA networks (Fig. 2B and Supplementary Fig. S3). We found that RNA-RNA interactions shared more miRNAs have higher correlation coefficients (Fig. 2C and Supplementary S4). In addition, we also found that RNA-RNA pairs with high degree (in the top 10%) were more highly co-expressed than pairs with low degree (degree = 1 and 2) (Fig. 2D and Supplementary Fig. S5). Next, we performed modularity analysis on the ceRNA networks, identifying modules using multi-level optimization of modularity in the R package “igraph”. With increasing of community size, the number of communities significantly decreased (Fig. 2E and Supplementary Fig. S6). This result suggests that ceRNAs exert specific functions in CVDs as small modules rather than as individual or large modules.

Evidence supports that expression levels of miRNAs determined the activity of ceRNA cross-talks^27^. We then investigated the effect of miRNA expression on ceRNA cross-talks in each CVD. Dicer, a crucial enzyme in miRNA-processing, is important in miRNA maturation. Here, we divided our samples into two groups, Dicer-low and Dicer-high, based on Dicer expression from profiles. In most datasets, co-expression patterns were more obvious in Dicer-low groups compared to Dicer-high groups (Fig. 2F and Supplementary Fig. S7). We infer that a medium concentration of miRNAs is necessary to maintain ceRNA cross-talk and abundant miRNAs could lead to the degradation of most genes. This result may be used to identify miRNA mediated ceRNA interactions.

### Network analysis highlights a common ceRNA network

**Figure 3:**
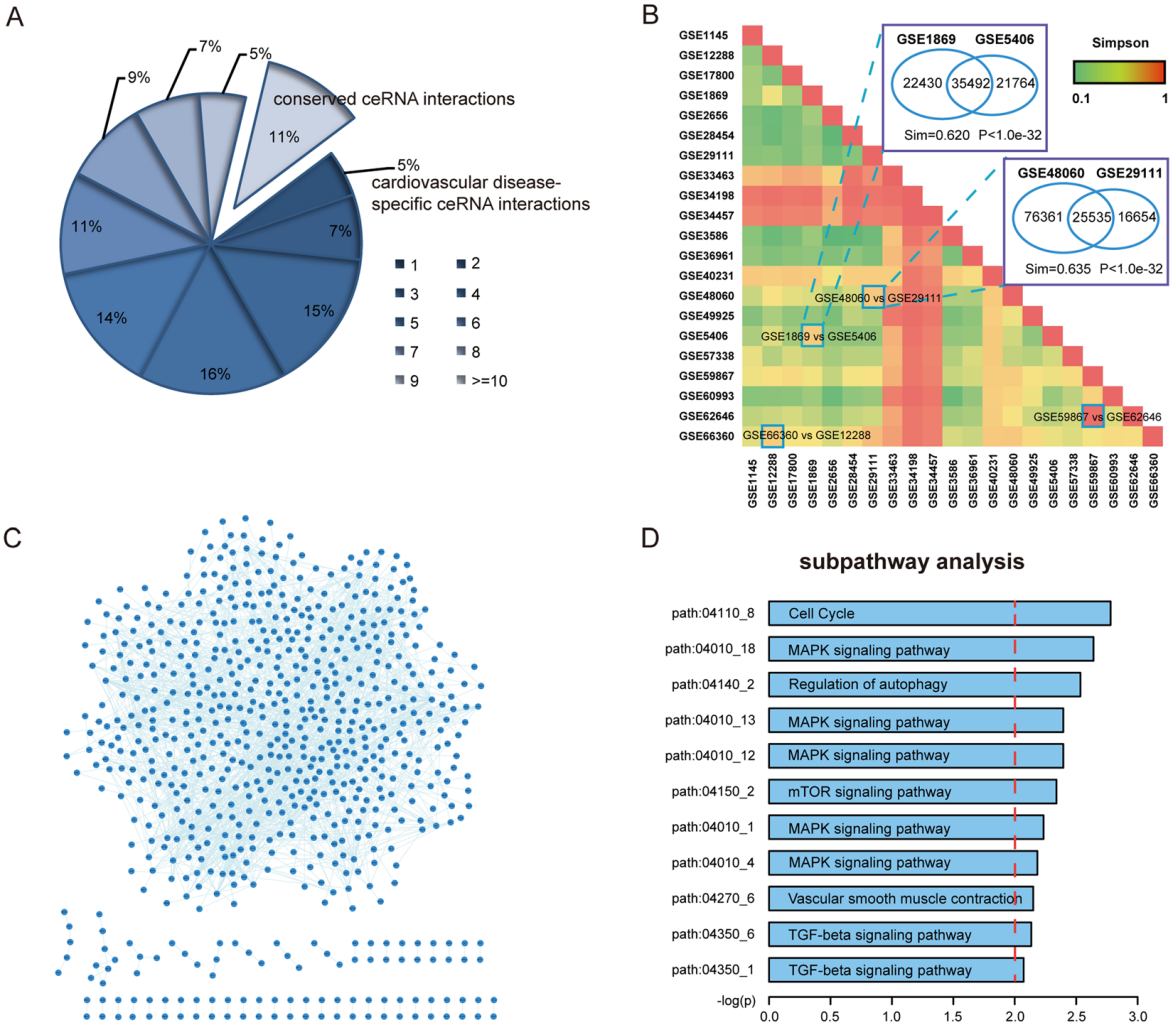
Comparison analysis of CVD-related ceRNA networks. (**A**) The pie chart of the distributions of ceRNA interactions across CVDs. A large number of ceRNA interactions were conserved. (**B**) Simpson index matrix represented the similarity between each pair of ceRNA networks. CeRNA networks belonging to same disease were more similar than networks not in the same disease. (**C**) A common core ceRNA were extracted in more than 15 profiles. (**D**) Subpathway enrichment analysis of ceRNAs in the core ceRNA network.

After analysis of the common features of ceRNA interactions, we compared ceRNA networks in diverse profiles and highlighted a common ceRNA network that may be important for ceRNA cross-talk in CVDs. Interestingly, we found that only 5% of ceRNA interactions appeared in one profile and ~10% of interactions were implicated in more than 10 profiles (Fig. 3A). This result indicates that ceRNA interactions are highly shared among CVDs and are important in multiple CVDs.

Because of the different distributions of common the ceRNA interactions, we performed Simpson index to measure similarities between profiles. We found that ceRNA networks associated with the same CVD, from highly correlated CVDs or from the same microarray platforms shared more ceRNA interactions than others (Fig. 3B). Based on the Simpson indexes, GSE48060 (MI, GPL570) and GSE29111 (MI, GPL570), GSE59867 (MI, GPL6244) and GSE62646 (MI, GPL6244) shared more ceRNA interactions than others (Sim = 0.635, p < 1e-32 and Sim = 0.962, p < 1e-32, respectively). For high correlated CVDs, the GSE1869 (ICM, GPL96) and GSE5406 (HF, GPL96) pair had got a high Sim score (Sim = 0.62, p < 1e-32). Cardiac muscle with long-time ischemia can lead to cardiac hypertrophy and fibrosis and heart failure. Similarly, for the GSE66360 (MI, GPL570) and GSE12288 (CAD, GPL96) pair, with Sim = 0.616, p < 1e-32. One of the main causes of myocardial infarction is coronary artery blockages. This could block blood flow and lead to cardiac ischemia and local inflammation. We found that two networks (GSE34198, GSE34457) from the same platform shared more interactions than others and we presumed that the platform caused this result. A common core ceRNA network component was observed in more than 15 networks across CVDs and was identified for further analysis (Fig. 3C). Finally, we performed sub-pathway enrichment analysis for genes from the ceRNA core component (Fig. 3D and Supplementary Table S3). Results showed that many CVD-related pathways were enriched, such as cell cycle, MAPK signaling pathway, regulation of autophagy and TGF-beta signaling pathway. This result indicates that the core ceRNAs may conduct their regulatory functions by involving in these pathways.

### Differential analysis of networks reveals multi-type hubs in each disease network

**Figure 4:**
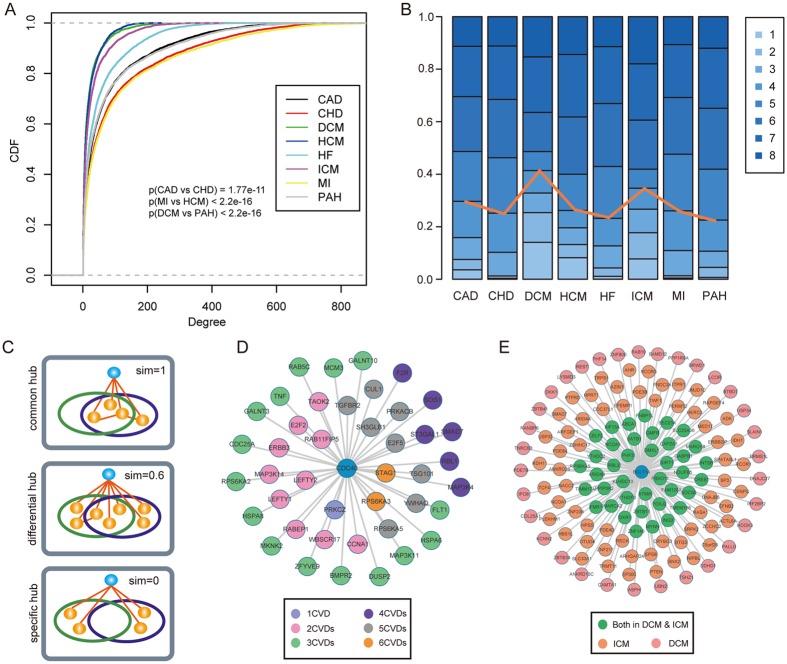
Differential analysis of networks reveals multi-type hubs in each disease network. (**A**) Cumulative distribution functions of the ceRNA degree in each disease network. (**B**) Hub ceRNA distributions across eight CVD ceRNA networks. (**C**) Hub ceRNAs were grouped into three categories, common hubs, differential hubs and specific hubs, showing in the mimic diagram. (**D**) An example of common hub CDC40. Nodes with different colors represented the different numbers of CVDs. (**F**) An example of differential hub BCL11A.

Next, we analyzed topological features of ceRNAs in multiple CVDs to reveal the common or specific mechanisms in biological processes. The 21 ceRNA networks of gene expression profiles that belonged to eight diseases were merged into eight ceRNA networks of diseases based on disease classification. The degree cumulative distribution has been performed to each disease network, showing that all networks had many genes with few interactions and a small subset of genes, defined as hubs, linked many genes (Fig. 4A). Based on the results of Kolmogorov-Smirnov tests, we found a significant change of degree distributions between different networks, such as the MI and the HCM networks (p < 2.2e-16), the CAD and CHD networks (p = 1.77e-11). These results suggest that ceRNAs have diverse connectivity features across CVDs, encouraging us to apply connectivity based measures to identify the crucial ceRNAs in CVDs. A single ceRNA with different degree in multiple networks might have different effect on cardiovascular biology, driving us to develop a measure to investigate them in depth.

Previous studies found that hubs with high degrees in biological networks are crucial for complex regulatory processes^28^. Thus, we identified hubs for each disease network. After comparing hubs across CVDs, we found that a large number of hubs occurred in more than one CVD (Fig. 4B). For example, only 3.6% of hubs were specific to the CAD ceRNA network, more than 50% of hubs in the CAD ceRNA network were hubs in more than five CVDs. These results suggest that most ceRNAs retain high connectivity across CVDs. To systematically analyze the extent of hubs sharing between different networks, we grouped hubs into three categories, common hubs, differential hubs and specific hubs (Fig. 4C). As a result, we identified 753 common, 106 differential and 192 specific hubs, with common hubs having the largest proportion. For example, the ceRNA CDC40 occurred in six disease networks as a hub and 10 ceRNA neighbors were shared in more than four networks (Fig. 4D). In addition, we performed sub-pathway enrichment analysis for all these ceRNAs and enriched the pathways (Supplementary Table S4), such as TGF-beta signaling pathway, Cell cycle and MAPK signaling pathway, indicating that these ceRNAs implicated in the processes of CVDs by the same mechanism. Next, we tested if the differential hubs had different regulatory roles in CVDs. Interestingly, we found a differential ceRNA BCL11A (Fig. 4E), which was a hub in the network of ICM and DCM, sub-pathway enrichment of ceRNA partners of BCL11A in ICM found that the TGF-beta signaling pathway and Axon guidance were significant. However, enrichment analysis of ceRNA partners in DCM showed that these genes were involved in the Wnt signaling pathway and the common partners were enriched in TGF-beta signaling pathway, Cell cycle and Jak-STAT signaling pathway (Supplementary Table S5). Overall, our results suggest that the roles of hub ceRNAs may exert functions by interacting with neighbors in CVDs.

### ceRNA regulations in CVD-related pathways

**Figure 5:**
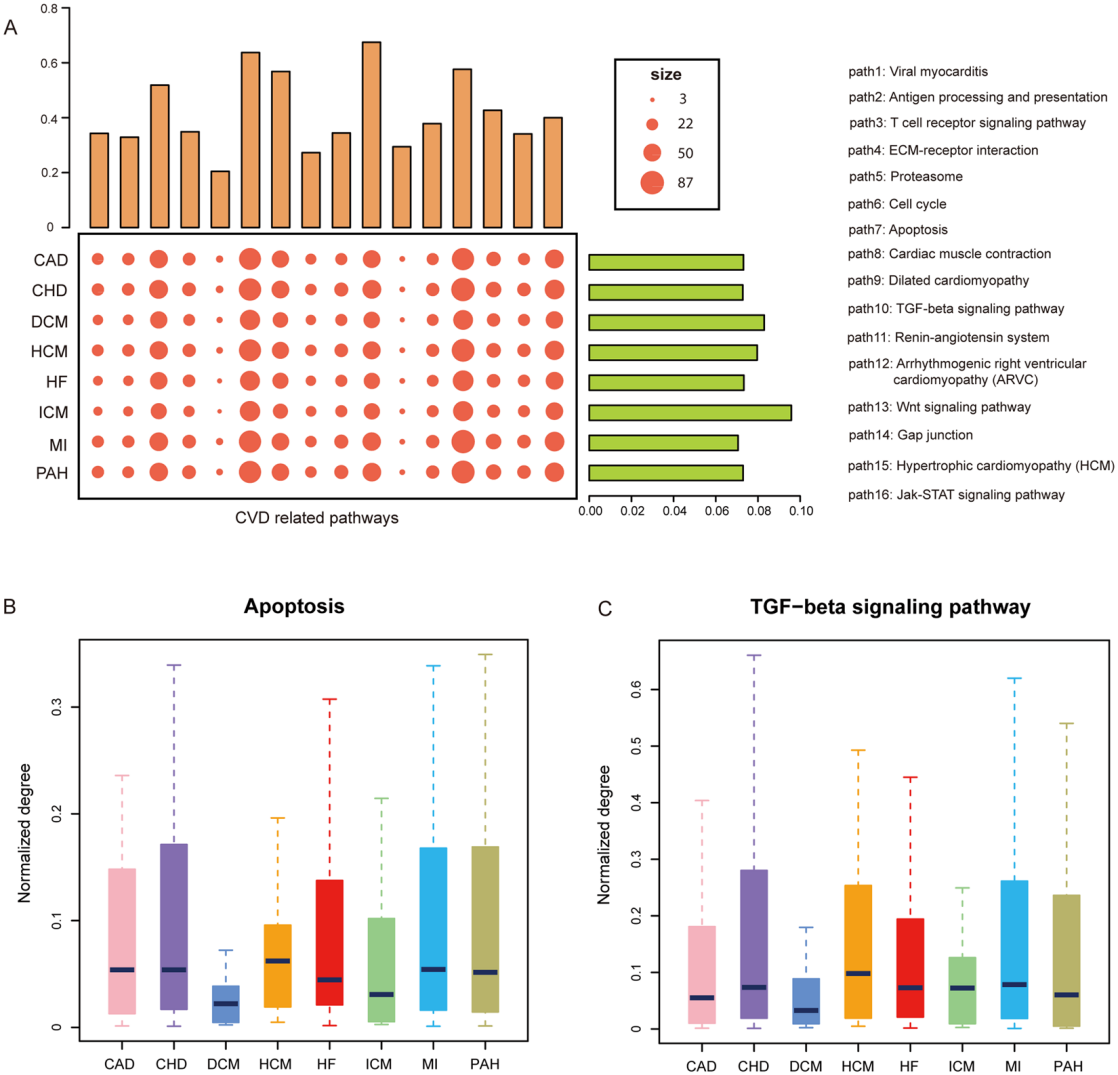
CeRNA regulations in CVD-related pathways. (**A**) The bubble and bar diagram showed the enrichment results of the CVD-related pathway genes in ceRNA networks across CVDs. The top bars showed the percentage of ceRNAs in CVD networks mapped in each CVD-related pathway and the right bar showed the percentage of ceRNAs in CVD-related pathways mapped in each CVD network. (**B**) The normalized degree of ceRNAs mapped in “Apoptosis”. (**C**) The normalized degree of ceRNAs mapped in “TGF-beta signaling pathway”.

The KEGG database collected cardiovascular diseases pathways as a category and we extracted genes from CVD-related pathways. These pathway-associated genes shed new light to the mechanism of ceRNA genes in different CVDs. As a result, 996 genes were extracted from 16 CVD-related pathways. Then we mapped all these genes of CVD-related pathways into the eight disease networks and the distribution results were presented in Fig. 5A. From the mapped results, we found that most pathway genes were involved in ceRNA regulations, which suggests that pathway genes may play important roles in the cross-talks. On the other hand, different distributions were observed for different pathways. For example, more genes from Cell Cycle and Wnt signaling pathways were mapped than genes from other pathways, indicating that CVDs were more regulated by these pathways, as seen in other studies. In addition, all the CVDs showed the similar distributions, which revealed the similar mechanism in the processes of biology.

Next, topological analysis of CVD-related pathway genes was performed. Degree distributions were presented in Fig. 5B,C and Supplementary Fig. S8. The overall degree distributions of genes in each pathway were similar, suggesting the similarity in the disease networks. In pathways, such as the apoptosis pathway and TGF-beta signaling pathway, degrees of pathway genes were higher in CHD and MI compared to other diseases. These results indicate different strengths of pathway genes in different CVDs. These features of CVD-related pathway genes could be used to identify more additional genes involved in the cardiology based on the ceRNA cross-talks.

### Analysis of ceRNA networks reveals multiple common ceRNA modules in CVDs

**Figure 6:**
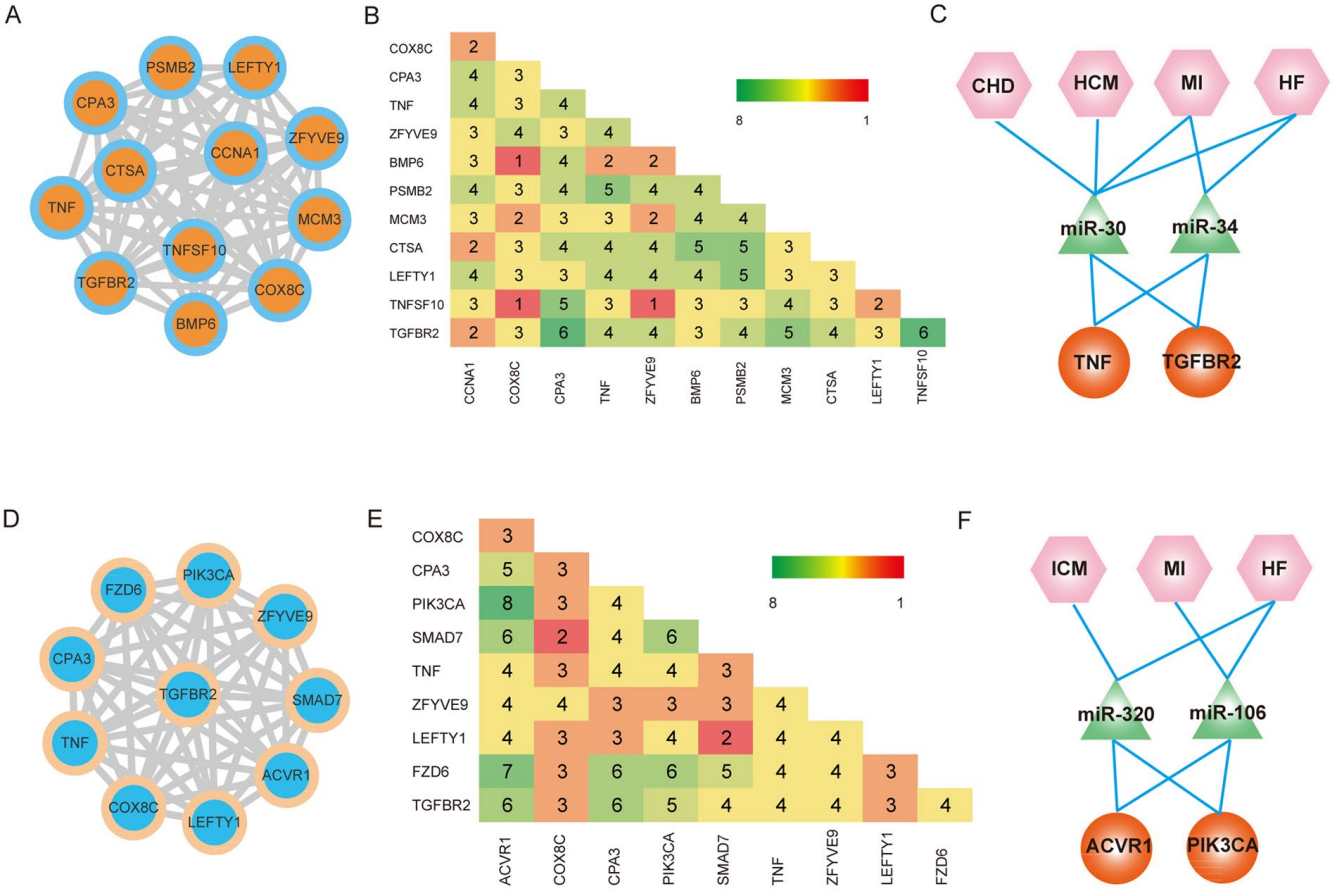
Analysis of ceRNA networks reveals common ceRNA modules. (**A**) An example of common module encompassed 11 ceRNAs. (**B**) The distribution matrix represented the number of CVDs which each ceRNA pair occurred. (**C**) The potential mechanism of ceRNA cross-talks in multi-CVDs. (**D**–**F**), Another example of common module encompassed 9 ceRNAs.

To explore the potential function of pathway genes in the regulatory mechanisms of CVDs, we were interested in identifying common and specific modules, because most genes exert their functions through interacting with each other. First, we merged all disease networks into a global CVD-related ceRNA network and mapped all CVD-related pathway genes into this network. As a result, a connected ceRNA network that contained pathway genes was extracted for common module identification. Modules in the network represented groups of genes with similar functions. In total, 131 common modules were identified (Supplementary Table S6). Functional analysis of these modules revealed that ceRNAs in these modules were involved in multiple CVD-related pathways. For example, a common module that encompassed 55 ceRNA pairs and 12 ceRNAs is in Fig. 6A–C. TNF, TGFBR2 and CCNA1 in the module played crucial roles in the biology process of multiple CVDs. Formation and release of TNF and its downstream signal transduction cascade is involved in the pathogenesis and progression of atherosclerosis, myocardial ischemia/reperfusion injury and heart failure^29^. Over-expression of TNF could reduce the expression level of PGC-1 by activating p38 MAPK and NF-κb signaling pathways, which could lead to heart failure^30^. Interestingly, previous studies found that TNF-α played important role in cardiac dysfunction because it increased the expression of TGF-β, and TGF-β receptors type I and II^31^. In addition, several miRNAs, such as miR-30 and miR-34, which mediate cross-talk between TNF and TGFBR2 were demonstrated to be dysfunctional in multi CVDs. Over-expression of miR-30 could decrease the expression level of CTGF induced by TGF-β and decrease production of collagens in cardiac fibrosis^32,33^.

Another common module contained 36 ceRNA pairs and 10 ceRNAs, including the known CVD-related genes ACVR1, PIK3CA and SMAD7 (Fig. 6D–F). Studies found that PIK3CA could regulate an L-type Ca^2+^ channel in cardiomyocyte, and reduced PIK3CA could lead to the decreased numbers of L-type Ca^2+^ channels and attenuated contractile dysfunction^34^. For cross-talk of ACVR1 and PIK3CA, we found that miR-320 and miR-106 were key molecules in the processes of CVDs. To identify specific modules, we mapped all these CVD-related pathway genes into each disease network and performed a module identification algorithm to these networks. Only a few ceRNA pairs were extracted from each network and no module was identified, which suggests that CVD specific hubs are not linked closely in networks. These results indicate that the cross-talks between CVD pathway-related common hubs played important roles in the mechanism of multiple CVDs by targeting upstream known disease-related miRNAs and exerted their functions within close modules.

### ceRNA cross-talk associated with pathways

**Figure 7:**
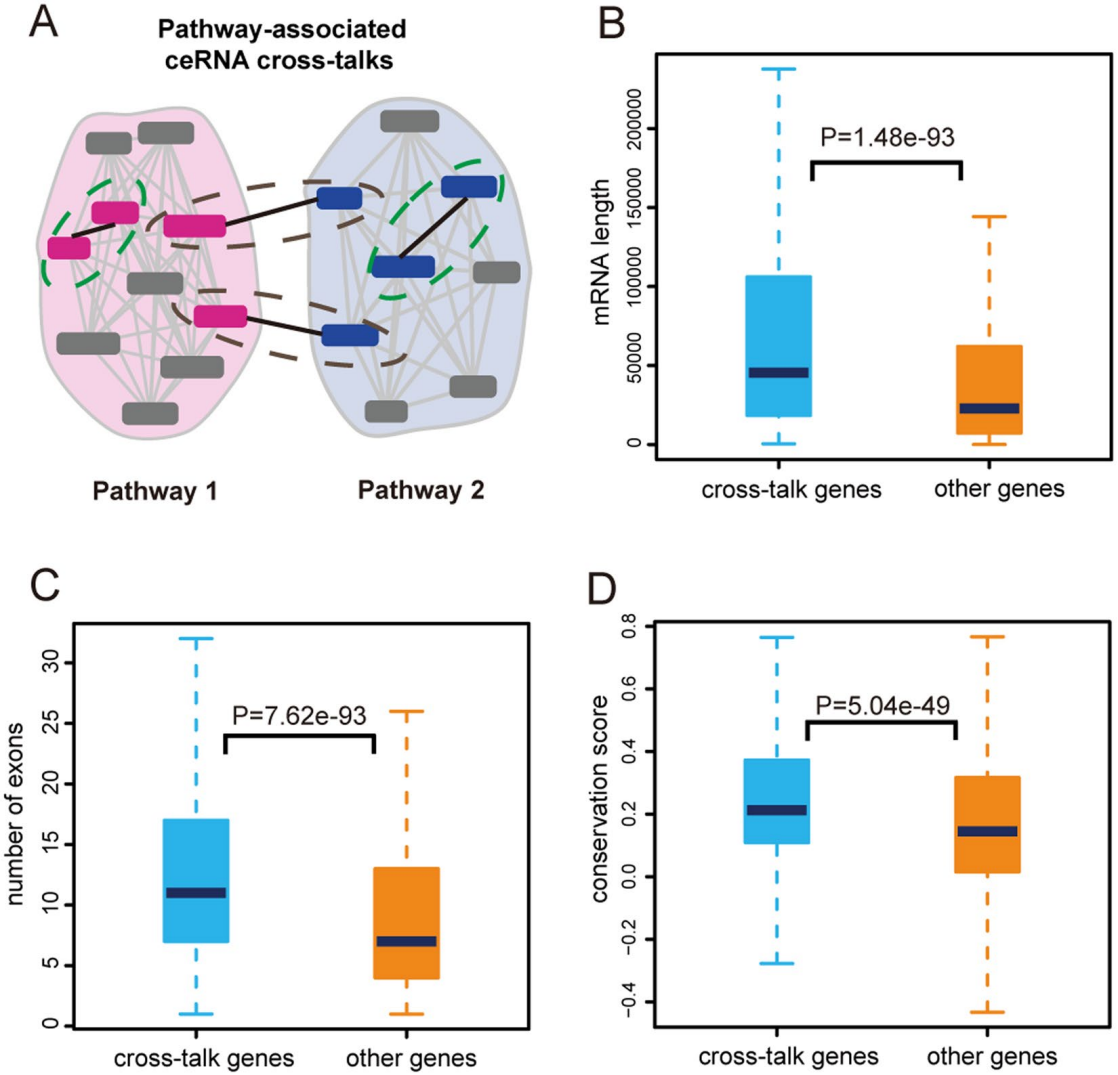
Pathway-associated ceRNA cross-talks. (**A**) The mimic diagram of two types of pathway-associated ceRNA cross-talks. Dotted green circle represented CPWP and dotted gray circle represented CPBP. (**B**) Comparison analysis of transcripts lengths between groups of cross-talk genes and other genes. (**C**) Comparison analysis of number of exons between groups of cross-talk genes and other genes. (**D**) Comparison analysis of conservation score between groups of cross-talk genes and other genes.

After analysis of ceRNA cross-talk in CVD-related pathways, we then investigated the properties of ceRNA cross-talks between or within pathways (Fig. 7A). Some studies found the presence of pathway cross-talks. For instance, studies had found that TGFβ could activate p38/MAPK, leading to the change of the ratio of apoptosis to proliferation in pulmonary arterial hypertension^35^, and PI3k could activate mTOR to regulate autophagy in cardiomyocytes^36^. We defined two types of ceRNA cross-talks, CPWP and CPBP (see in Materials and methods). As a result, we identified 37,332 ceRNA cross-talk pairs that were associated with pathways. We then split these ceRNA pairs into two groups for a total of 2,366 CPWPs and 34,966 CPBPs. Most ceRNA cross-talks were involved in CPBP, indicating that ceRNA might play an important role in pathway cross-talk, which was regarded as an important mechanism in disease development processes. Interestingly, some ceRNA belonged to most of pathways, such as the MAPK family, PIK3CA and TNF, which mediate important pathway cross-talks and are demonstrated to be known disease genes. Next, we investigated the properties of ceRNAs involved in these pathway-associated cross-talks and other genome genes. As a result, pathway-associated ceRNA transcripts were longer and had more exons than other transcripts (Fig. 7B,C). Moreover, pathway-associated ceRNA transcripts were more conserved than other transcripts (Fig. 7D). These results suggest potential regulatory roles for ceRNA cross-talks in pathway cross-talks and provide supports to uncover the pathological mechanism in pathway levels.

## Discussion

In our study, we performed a global analysis of mRNA-related ceRNA cross-talks in eight major CVDs from 21 gene expression profiles. Interestingly, some common properties were identified from these CVD-related ceRNA cross-talk networks. For instance, all these ceRNA networks followed power law distributions. The ceRNAs with a high degree and ceRNA pairs shared more miRNAs showed a more co-expressed feature. In addition, we found that co-expression patterns were controlled by miRNA expression levels. Comparison of ceRNA interactions across diverse ceRNA networks identified some conserved interactions and a common core ceRNA network were extracted, which suggests that a fraction of ceRNAs may regulate biological processes involved in CVD-related pathways, such as Cell Cycle and MAPK signaling pathway. It is well known that hub nodes are topologically centered and play key roles in biological regulatory networks. We performed a systematic analysis of the hub ceRNAs from each disease network and grouped them into three categories, common hubs, differential hubs and specific hubs. These hubs were used to uncover the pathological mechanisms of ceRNA interaction in CVDs. Importantly, mapping CVD-related pathway genes into the merged ceRNA network identified ~130 common modules, but no specific modules. This result suggests that common hub ceRNAs closely linked and might function in common modules. Finally, we investigated the relationship between ceRNA cross-talks and pathway cross-talks. Most ceRNA interactions participated in many pathway cross-talks and pathway cross-talk.

miRNA mediated mRNA-related ceRNA cross-talks have obtained more attentions nowadays. Many studies have found that this type of ceRNA cross-talk is present in diverse diseases, such as cancers and immunological diseases. For instance, in cancers, Xu *et al*. provided a landscape of ceRNA interactions across 20 major cancers^23^. By comparing of the results between cancers and CVDs, common features have been proposed. First, both cancer and CVD-related ceRNA networks followed a power law distribution, composed of small size modules and miRNA expression levels that directly determined the activity of ceRNA cross-talk. These results suggest that ceRNA might function with similar regulation patterns. And we found that common ceRNA interactions in cancers and CVDs were involving in some common pathways, such as Cell Cycle and MAPK signaling pathways. This result indicates similar signaling mechanisms between cancers and CVDs. In addition, analysis of hub ceRNAs showed that common hubs accounted for a large population of hubs in both cancers and CVDs. However, several CVD specific features were also been identified. Pathway enrichment analysis of common ceRNAs of CVDs showed that some pathways were only significantly enriched in CVDs, such as mTOR signaling pathway and Regulation of autophagy. This result suggests that the mechanisms of ceRNAs in CVDs are also associated with these pathways. For identifying the specific modules, we mapped CVD-related pathway genes and specific hubs into each disease network. No specific module was identified. This result suggests that specific hubs were not linked closely in networks.

In recent years, more and more studies have focused on the complex regulatory patterns of miRNAs in multiple diseases. And studies have found that miRNAs play key roles in the biology processes of CVDs. Our systematical analysis of miRNA mediated mRNA-related ceRNA cross-talks also contributed to elucidate the potential mechanism of miRNA in CVDs. For example, we highlighted a common core ceRNA networks across multiple CVDs, which could reveal similar mechanism among CVDs. Importantly, we found that many common modules composed a large regulatory network and functioned in many CVDs. For the ceRNA pairs of TNF- TGFBR2 in a common module, many studies have demonstrated the relationship between them in CVDs. For instance, TGF-beta signaling pathways might suppress PPAR-α activity in cardiac myocytes by locally elevating TNF in cardiomyopathy^37^, and TNF-α played important role in cardiac dysfunction by increasing the expression of TGF-β, and TGF-β receptors type I and II^31^. In addition, this ceRNA pair was mediated by miR-30, which was involved in TGF-β pathway in CVDs^32,33^. These results suggest that the complete triplet encompassing ceRNAs and miRNAs is crucial in complex regulatory processes and provide a new mechanism for uncovering regulation patterns, such as activation or inhibition. Another interesting result was that ceRNA cross-talks could mediate pathway cross-talks. Many studies have found cross-talks between pathways in diverse diseases^38,39^, and some studies investigated the pathway cross-talks by integrating miRNA regulatory network^40^. In our study, we proposed a new level to elucidate pathway cross-talk. CeRNAs could compete for the shared miRNAs and then lead to the activation of pathways.

However, our present study had some limitations. For instance, no database, such as TCGA, is established for CVDs for storing high-throughput data to investigate molecular interactions. We performed the algorithm of hypergeometric test and Pearson correlation coefficients to identify miRNA sponges. This pipeline was a popular strategy that has been applied in many ceRNA researches, but also created false positive. The strategy could be improved to identify ceRNA cross-talks more accurately. Moreover, we integrated 21 gene expression profiles of eight CVDs from GEO database, but these expression profiles came from multiple platforms. Thus our results might be biased in some diseases. Multiple gene expression profiles were tested for multiple diseases. Some CVDs have similar disease phenotype on the pathology, such as MI, CHD and ICM, so we also regrouped these diseases into 6 categories: IHD (ischemic heart disease, mixing MI, CHD and ICM into one disease), CHD, DCM, HCM, HF and PH. We re-analyzed all these disease related ceRNA networks, including degree analysis and hub distributions. Results showed a high similarity compared with results of 8 disease related ceRNA networks. For example, we performed degree cumulation distributions for each disease network (Supplementary Fig. S9), showing that all networks had many genes with few interactions and a small subset of genes, defined as hubs, linked many genes. Different disease network has different connectivity features. In addition, we identified hubs for each disease network and performed a hub distribution for all networks (Supplementary Fig. S10). A majority of hubs were shared more than one disease. These results indicated a similar biological phenomenon could be obtained from different category of CVDs.

In summary, our study analyzed global mRNA-related ceRNA cross-talks across eight CVDs and provided a new method to investigate the biology mechanism of CVDs. By integrating data sets encompassing a large number of samples from different CVDs could suggest common or specific features of ceRNAs and enable us to identify more available target genes in pathological processes. In addition, our study uncovered complex CVD-related biological processes by integrating genome data. This method could help to explore more unknown mechanisms implicated in CVDs.

## Methods

### Protein-coding gene expression profiles of major cardiovascular diseases

All protein-coding gene expression profiles were downloaded from Gene Expression Omnibus (GEO, http://www.ncbi.nlm.nih.gov/geo/). GEO is a public functional genomics data repository supporting MIAME-compliant data submissions. In total, our study investigated eight major CVDs: coronary artery disease(CAD), hypertrophic cardiomyopathy (HCM), dilated cardiomyopathy (DCM), ischemic cardiomyopathy (ICM), heart failure (HF), myocardial infarction (MI), pulmonary hypertension (PAH) and congenital heart disease (CHD) in 21 gene expression datasets (detailed description see in Supplementary Table S1). As for the profiles with raw expression values, we performed log2 transformed to the gene expression values for the subsequent analysis.

### CLIP-seq-supported miRNA-mRNA interactions

In this study, CLIP-seq-supported miRNA-mRNA interactions were downloaded from starBase V2.0 database^18^. In total, we downloaded 423,975 miRNA-mRNA interactions that contained 386 miRNAs and 13,802 mRNAs. Crosslinking and Argonaute (Ago) immunoprecipitation coupled with high-throughput sequencing (CLIP-Seq) could identify the genome-wide interaction of miRNAs and their targeting RNAs^41^. starBase V2.0 database is designed for decoding interaction network via integrating large-scale CLIP-Seq (HITS-CLIP, PAR-CLIP, iCLIP, CLASH) data. MiRNA targets of starBase V2.0 were predicted by five target predicted algorithms, including TargetScan, miRanda, Pictar, PITA, and RNA22.

### Genes in the KEGG pathways of cardiovascular diseases

KEGG PATHWAY (http://www.kegg.jp/kegg/) is a collection of manually drawn pathway maps representing our knowledge on the molecular interaction and reaction networks. The sub-catalog “cardiovascular diseases” collected by KEGG pathway database was used in this study. The sub-catalog had four pathways drawn in the in KEGG database directly; we selected the related pathways that occurred in each of the four pathway maps as the CVD-related pathways indirectly. In total, we obtained 16 CVD-related pathways. Genes annotated in the 16 pathways were extracted by the R package of “iSubpathwayMiner”^42^, which was developed from our research group.

### CVD-related miRNAs

We obtained the CVD-related miRNAs from three databases, including HMDD^43^, miR2Disease^44^, and miREnvironment^45^. Then we mapped these miRNAs to each CVD manually. In total, after combining these results, we collected 702 miRNA-CVD pairs.

### Construction of CVD-related ceRNA network

We used two principles to identify the ceRNA pairs in each profile (Fig. 1). On the one hand, the activity of ceRNA cross-talk increased as the number of shared miRNAs between one pair of ceRNA increased. We listed all candidate mRNA-mRNA pairs from miRNA-mRNA interactions. These were obtained from CLIP-seq-supported miRNA-mRNA interaction relationships from starBase. We then computed the number of shared miRNAs of each candidate mRNA-mRNA pairs. We removed the pairs with less than 3 common miRNAs. A hypergeometric test was performed to estimate the significance of the relationship of each mRNA-mRNA pair that shared common miRNAs. The p-value was measured as:

$$p-value=1-\sum _{i=0}^{r-1}\frac{(\begin{array}{c}t\\ i\end{array})(\begin{array}{c}m-t\\ n-i\end{array})}{(\begin{array}{c}m\\ n\end{array})}$$

where, m represented the total number of human genome miRNAs, t represented the number of miRNAs interacting with the mRNA1, n represented the number of miRNAs interacting with the mRNA2, and r represented the number of miRNAs shared between mRNA1 and mRNA2. All p-values that were computed by hypergeometric test were adjusted by the method described by Holm^46^. On the other hand, based on the mechanism of ceRNA, over-expression of one ceRNA could result in the increased expression of the other one ceRNA in ceRNA cross-talk. This result suggests that the expression of ceRNA pairs is positively correlated. Then we computed the Pearson correlation coefficients and corresponding p-values, FDR values for candidate ceRNA pairs in each of 21 CVD-related gene expression profile. All the candidate ceRNA pairs with adjusted p-value < 0.01 from hypergeometric test and R > 0, p-value < 0.01 from Pearson correlation coefficient test were identified as ceRNA–ceRNA interactions. The ceRNA network of each CVD was generated by merging all ceRNA–ceRNA interactions of each gene expression profile. In the networks, nodes represented mRNAs and edges represented co-expressed ceRNA pairs regulated through miRNAs. All these networks were visualized by Cytoscape^47^.

### Hub nodes analysis of ceRNA networks

Some studies have demonstrated that hub nodes with higher degrees in biological networks were more important than others. We selected the top 10% of nodes with the highest degrees as the hub nodes in this study. To analyze the distribution of hub nodes across all CVDs, we classified the hub nodes into three categories according to the strategy proposed by Xu *et al*.^23^ (1) CVD-specific hub nodes; 2) differential hub nodes; and 3) common ceRNA hub nodes. Specially, the hub nodes that only occurred in one ceRNA network were defined as CVD-specific hub nodes. Differential hub nodes were the hub nodes occurred in more than one ceRNA network but their neighbors changed between different diseases. And common ceRNA hub nodes, that was defined as the ceRNAs were hub nodes in more than one ceRNA with similar neighbors. For the first category, the ceRNAs only ranked in top 10% of highest degrees in one ceRNA networks, for the other two categories, we calculated the similarity of their interacting neighbors between pairs of ceRNA networks by the method of Simpson index. If a hub node with Simpson index > 0.8 for at least one pair of ceRNA networks, we assigned the hub node into the third category. Otherwise, we grouped the hub nodes into the second category.

### Construction of CVD pathway-associated ceRNA networks

First, we merged all 21 ceRNA networks of gene expression profiles into eight ceRNA networks of diseases according to the disease name. We obtained the genes from CVD-related KEGG pathways using “iSubpathwayMiner” and mapped all the genes into the ceRNA networks of disease. Edges mediated by two KEGG pathway genes were extracted. Finally, CVD pathway-associated ceRNA networks were constructed.

### Identification of CVD-related ceRNA network modules

For each ceRNA network of gene expression profiles, we identified the modules by applying the method called multi-level optimization of modularity in R package “igraph”. This method was based on the modularity measure and a hierarchical approach. Modules that identified from the method were defined as communities^48^. We reserved the scale of community that was more than 3 and less than 300.

To identify modules of common hub ceRNAs in the CVD pathway-associated ceRNA networks, all the CVD pathway-associated ceRNA networks were merged and we extracted the sub-network edges linked by common hub nodes. We performed CFinder (http://www.cfinder.org/), a fast program for locating and visualizing overlapping, to find common modules defined as communities. Similarly, CVD-specific ceRNA modules were identified from the sub-network that composed of CVD-specific hub nodes for each CVD pathway-associated ceRNA network.

### Pathway-associated ceRNA cross-talk pairs

KEGG pathways were grouped into two categories: metabolic and non-metabolic pathways. We extracted genes from all the metabolic pathways and non-metabolic pathways using R package of “iSubpathwayMiner”. We proposed two types of ceRNA cross-talks associated with pathways: ceRNA pairs within pathways (CPWP), defined as one ceRNA pair only occurred in one pathway. Briefly, one ceRNA pair: gene1-gene2, both gene1 and gene2 occurred in pathway A. And the other one was that ceRNA pairs between-pathways (CPBP), which was defined as two ceRNAs that in one ceRNA cross-talk not occurred in same pathway. For example, one ceRNA pair: gene1-gene2, gene1 belongs to pathway A, gene2 not belongs to pathway A.

## Electronic supplementary material

Supplementary materials

Supplementary Table S6
