# Author Correction: The global view of mRNA-related ceRNA cross-talks across cardiovascular diseases

**DOI:** 10.1038/s41598-018-29002-8

**Published:** 2018-07-17

**Authors:** Chao Song, Jian Zhang, Hanping Qi, Chenchen Feng, Yunping Chen, Yonggang Cao, Lina Ba, Bo Ai, Qiuyu Wang, Wei Huang, Chunquan Li, Hongli Sun

**Affiliations:** 1 Department of Pharmacology, Harbin Medical University-Daqing, Daqing, 163319 China; 2 Department of Medical Informatics, Harbin Medical University-Daqing, Daqing, 163319 China

Correction to: *Scientific Reports* 10.1038/s41598-017-10547-z, published online 31 August 2017

The original version of this Article contained an error in the order of corresponding authors. This error has now been corrected in the PDF and HTML versions of the Article.

