# Supplementary material for: The global view of mRNA-related ceRNA cross-talks across cardiovascular diseases
Source: Sci Rep. 2017 Aug 31;7:10185. doi: 10.1038/s41598-017-10547-z (PMC5579186; doi:10.1038/s41598-017-10547-z)
Supplement: Supplementary file 1 — Supplementary materials [file 41598_2017_10547_MOESM1_ESM.pdf]

The global view of mRNA related ceRNA cross-talks across cardiovascular diseases

Chao Song[1, †], Jian Zhang[2, †], Hanping Qi[1, †], Chenchen Feng[2], Yunping Chen[1], Yonggang Cao[1], Lina Ba[1], Bo Ai[2], Qiuyu Wang[2], Wei Huang[1], Chunquan Li[2, *], Hongli Sun[1, *]

[1] Department of Pharmacology, Harbin Medical University-Daqing, Daqing, 163319, China

[2] Department of Medical Informatics, Harbin Medical University-Daqing, Daqing, 163319, China.

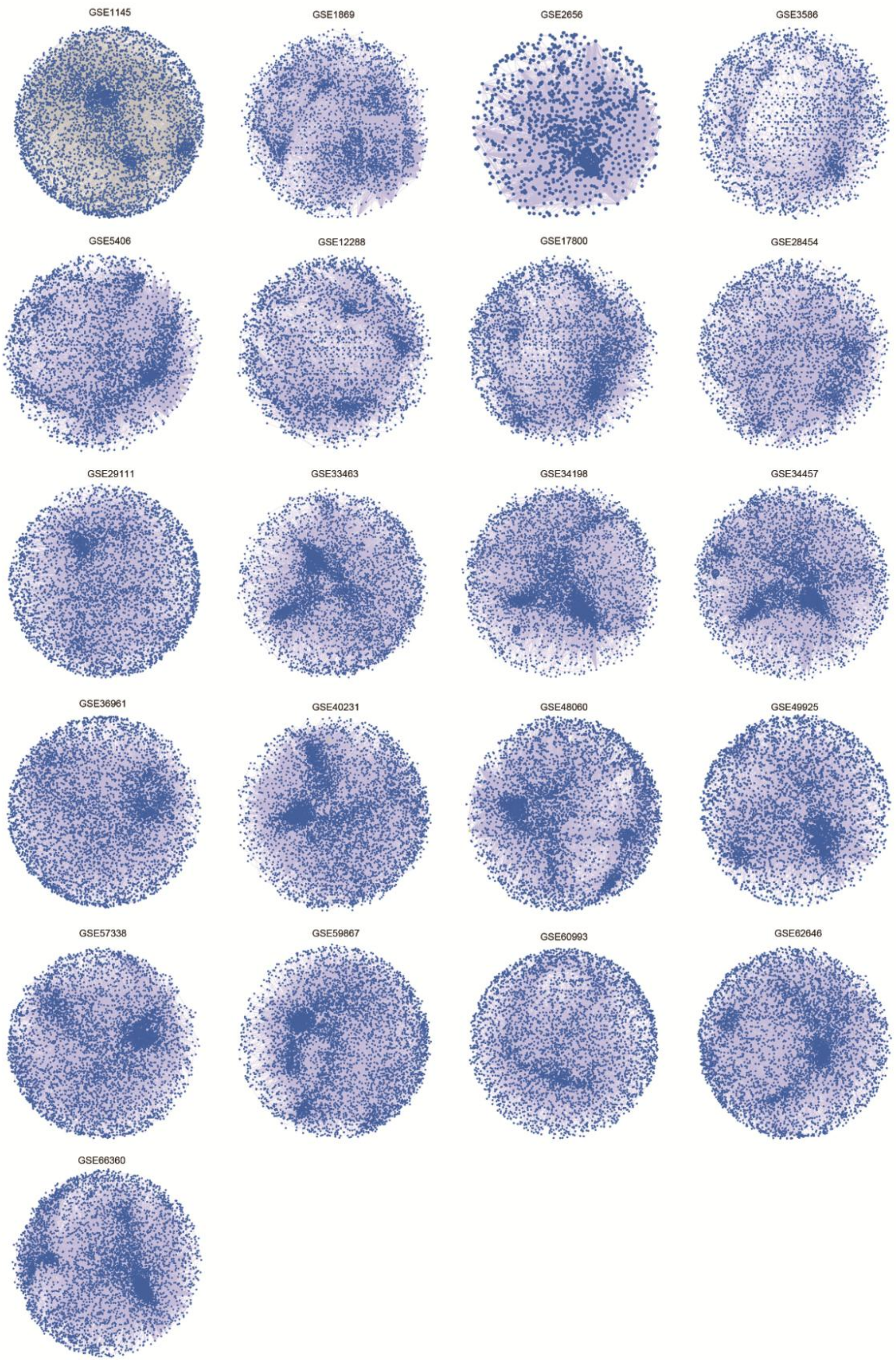

Supplementary Figure S1: The view of ceRNA networks. In our study, we performed 21 ceRNA networks from 8 CVDs and networks were visualized by Cytoscape.

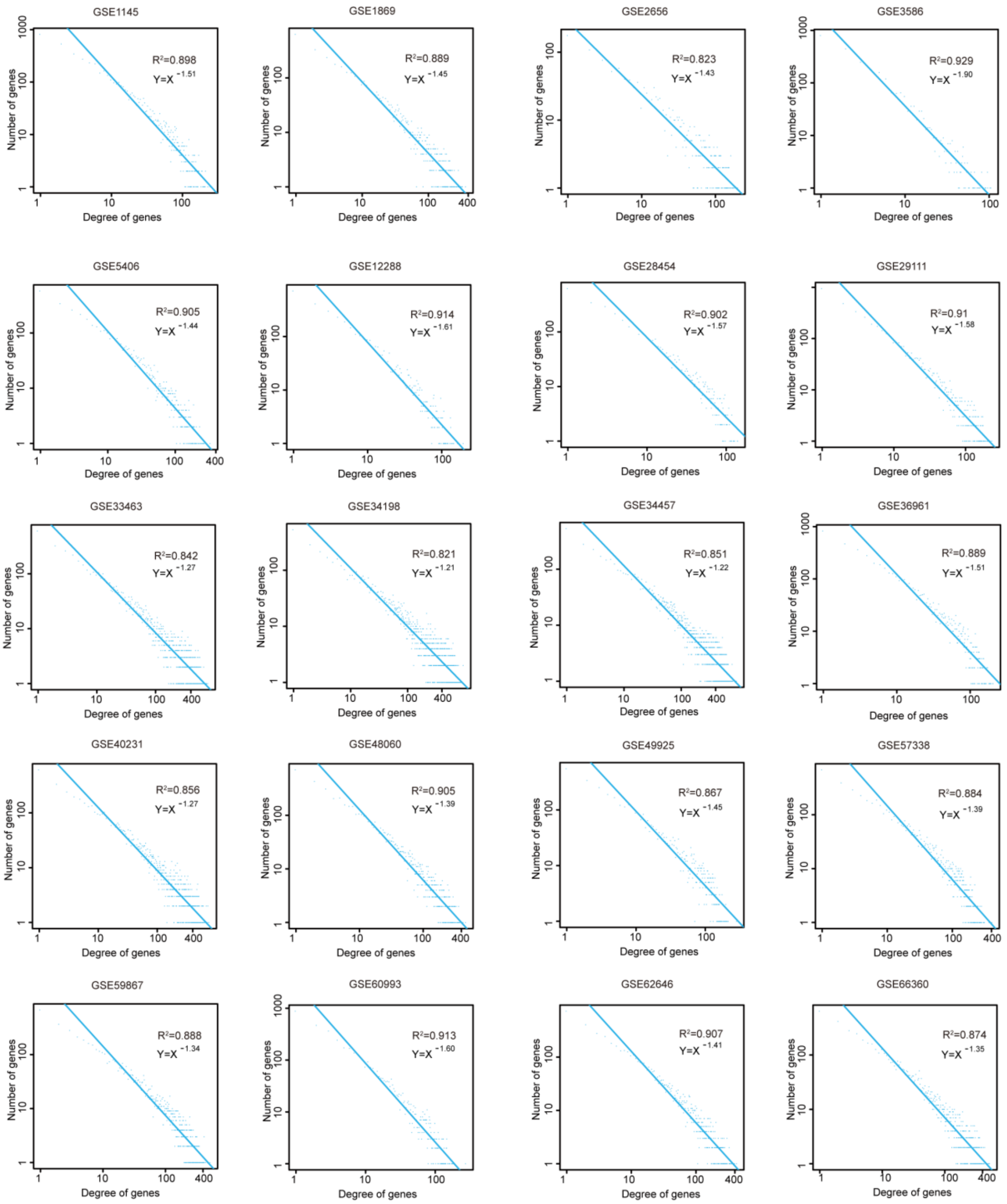

Supplementary Figure S2: Degree distributions of ceRNA networks. All these ceRNAs were followed power-law distributions, indicating that these ceRNA networks were scale-free.

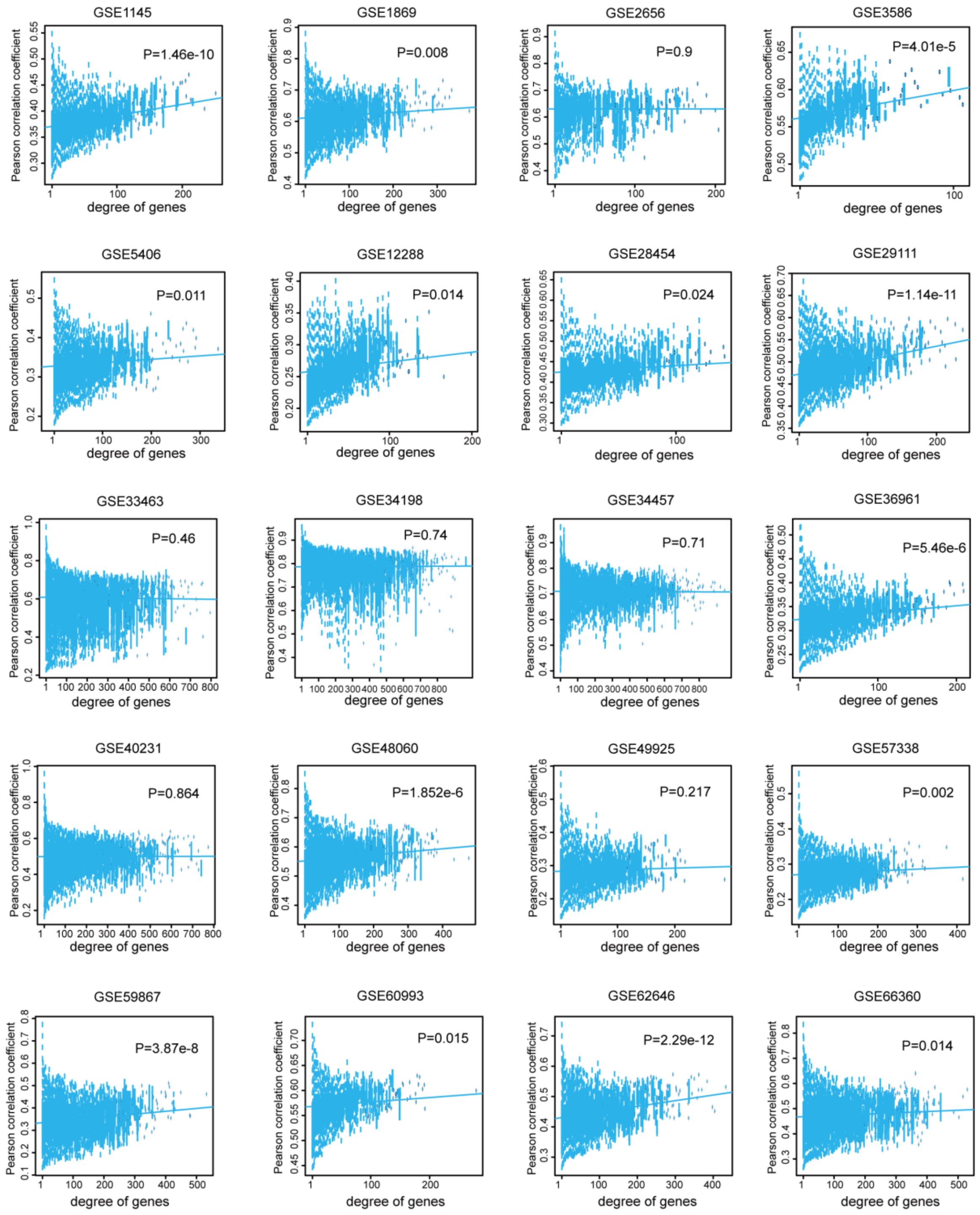

Supplementary Figure S3: ceRNAs with high degree were strongly co-expressed than others.

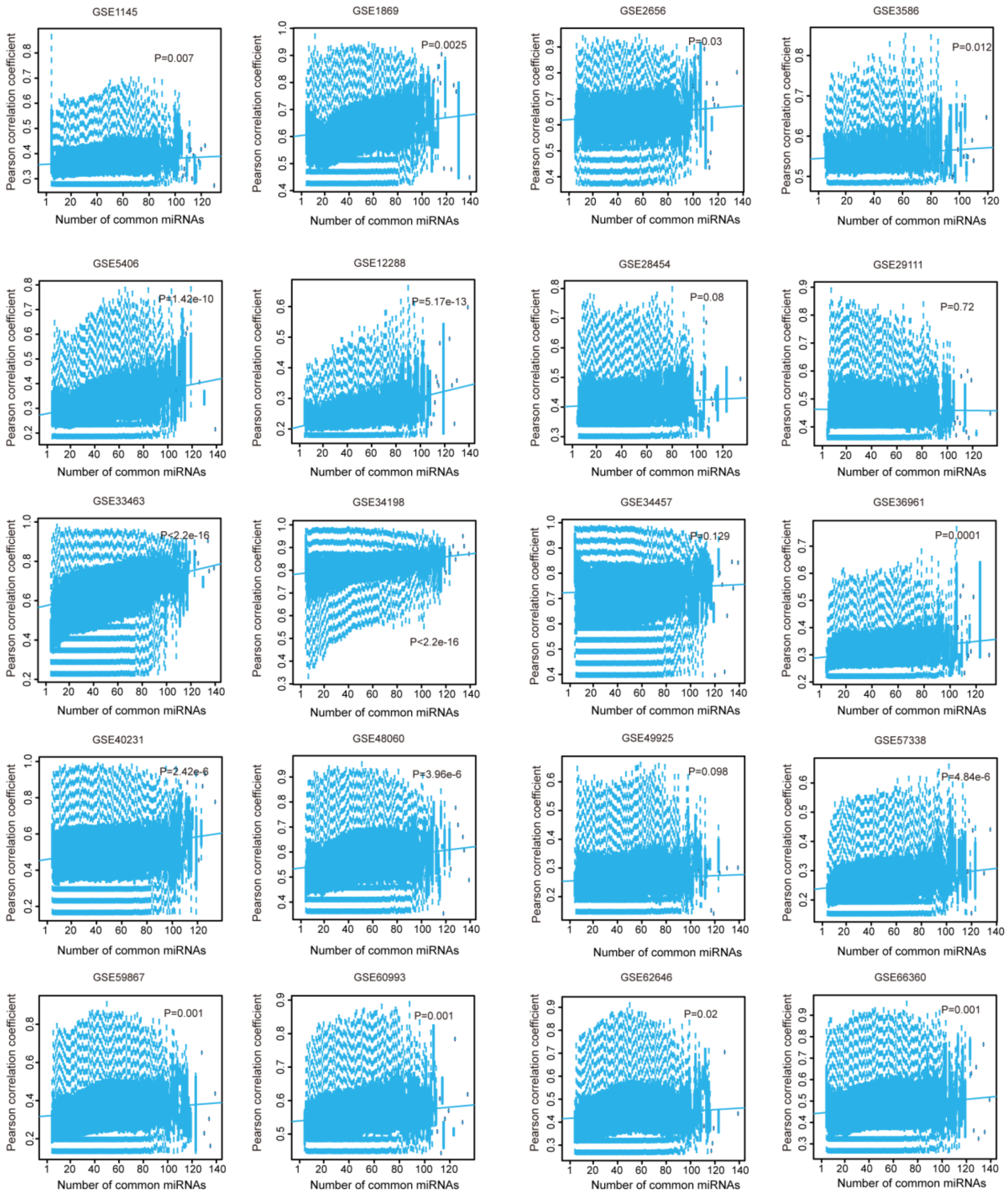

Supplementary Figure S4: ceRNA pairs shared more miRNAs were strongly co-expressed than others.

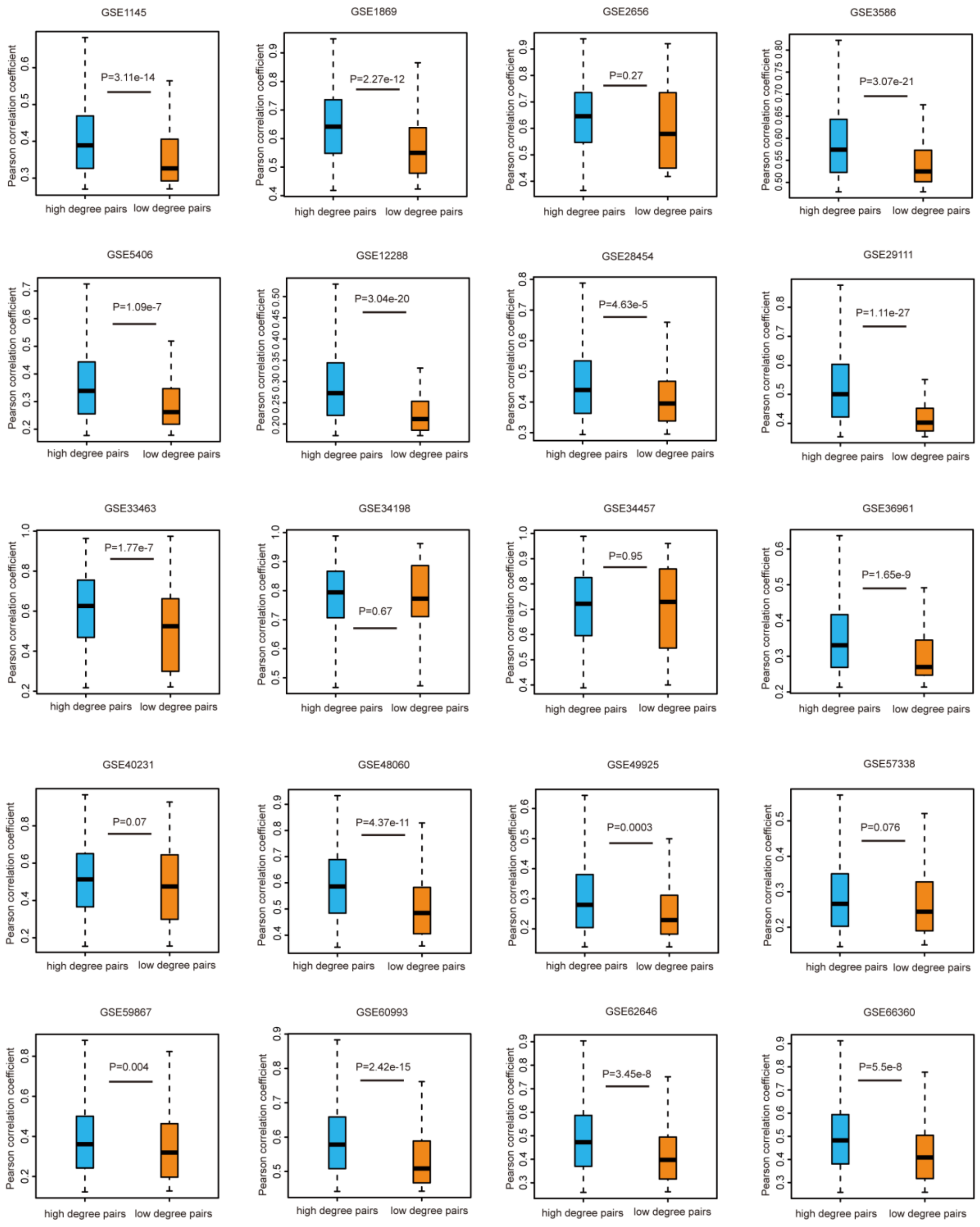

Supplementary Figure S5: ceRNA pairs with high degree were strongly co-expressed than others.

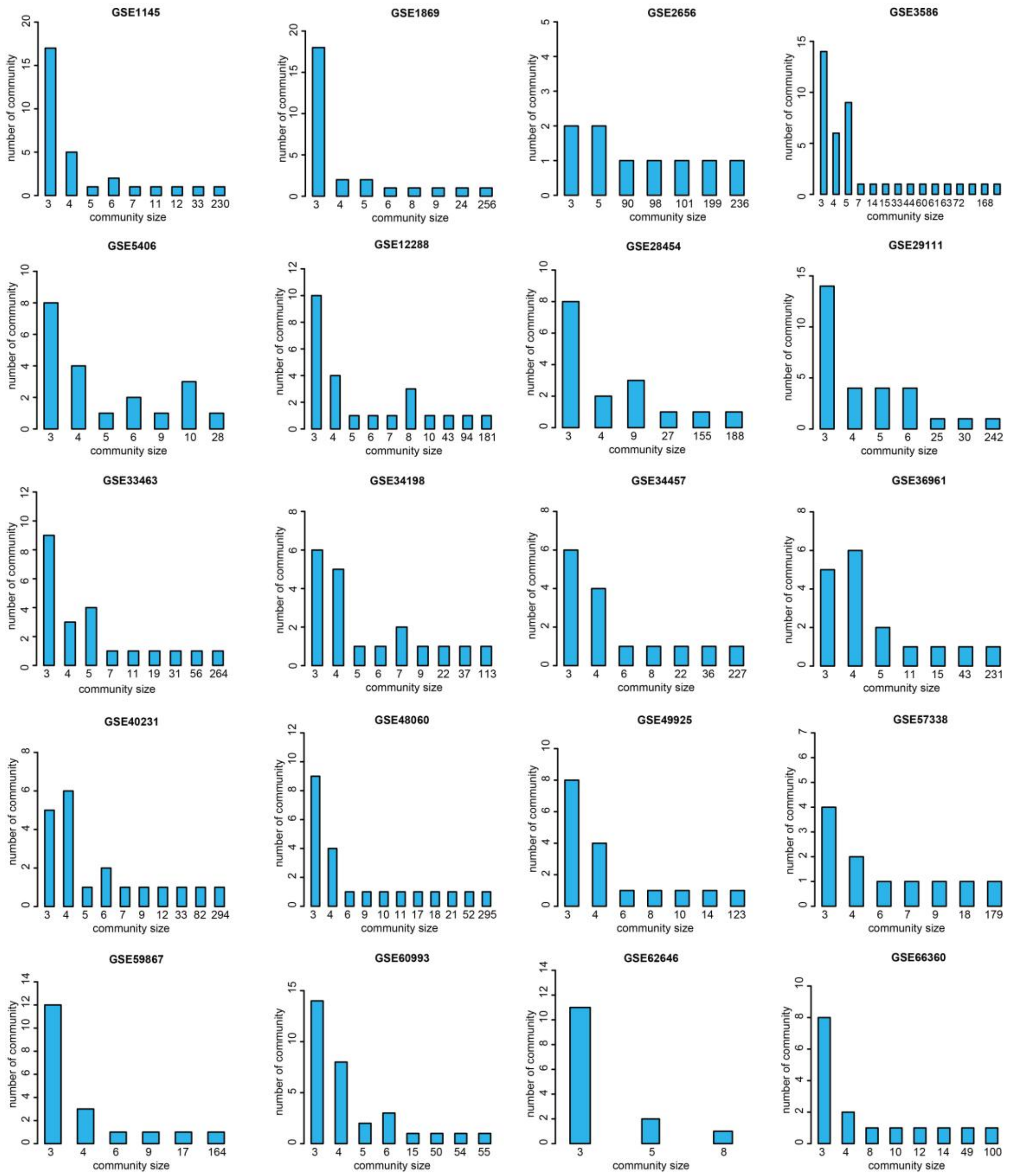

Supplementary Figure S6: ceRNAs were likely to exert functions in small communities.

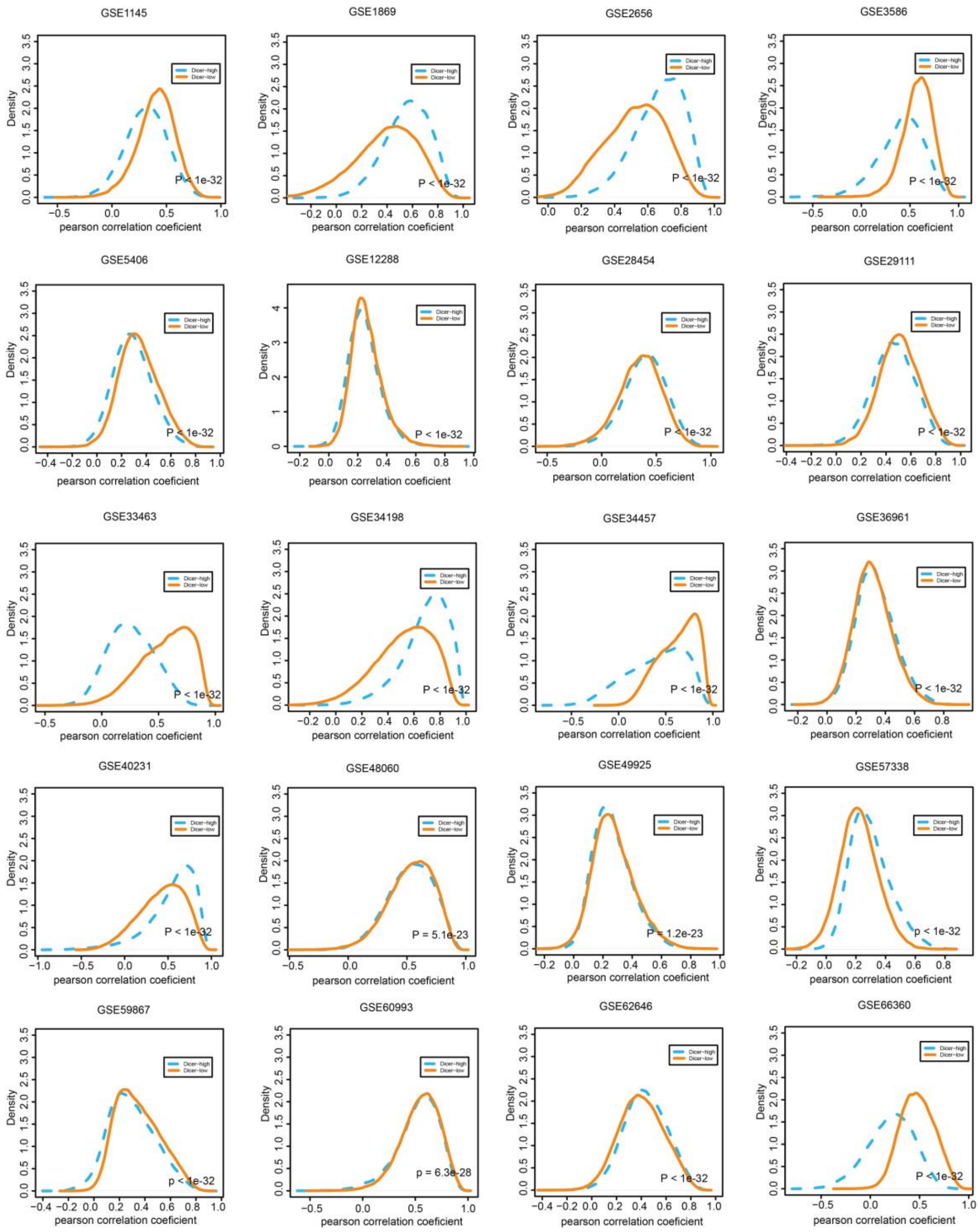

Supplementary Figure S7: ceRNAs were likely to co-express in Dicer-low groups.

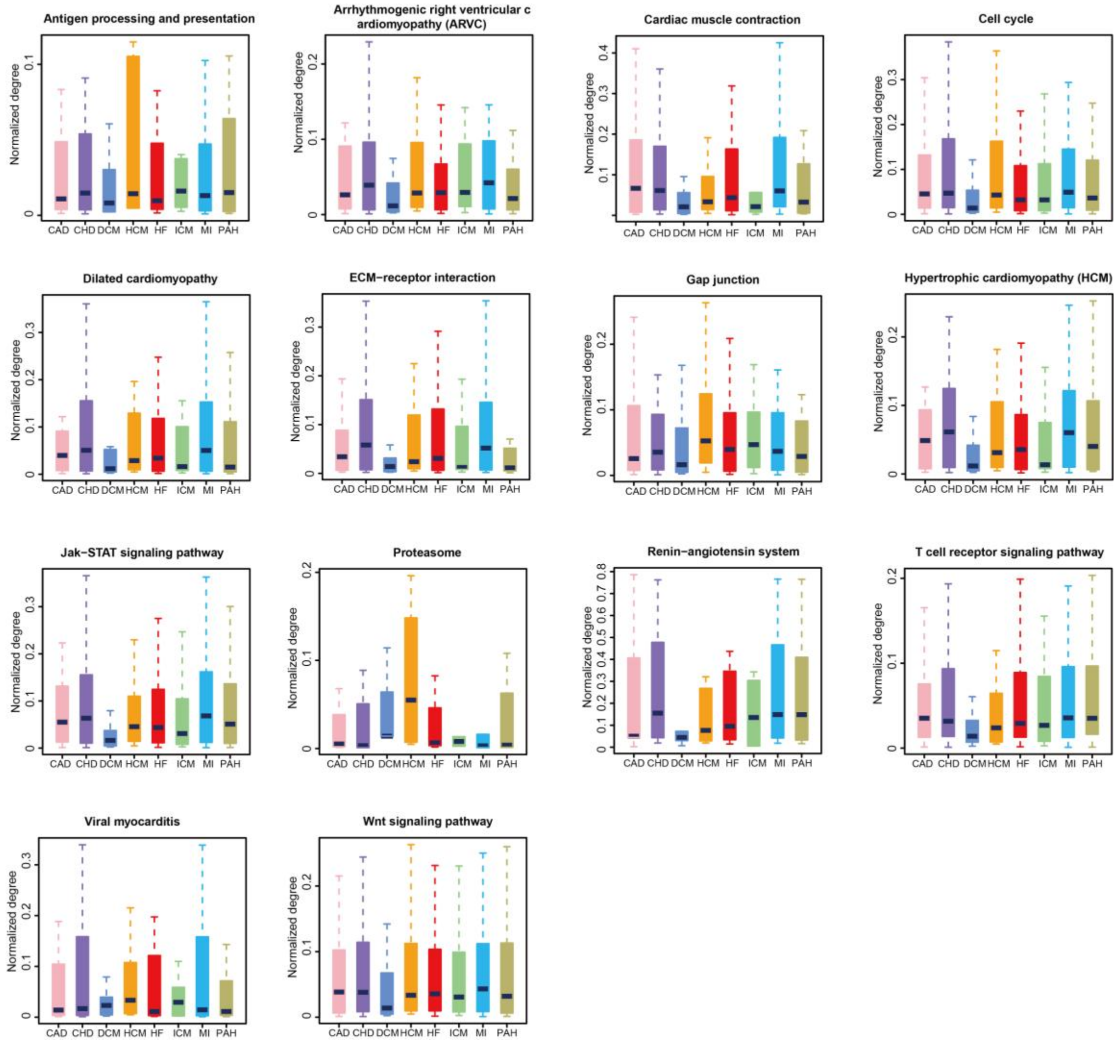

Supplementary Figure S8: Normalized degree of ceRNAs mapped in each CVD related pathways.

Supplementary Table S1

GSE	p-value	FDR	GSE	p-value	FDR
GSE1145	0.00999881	0.04279533	GSE36961	0.01	0.041233
GSE12288	0.00999893	0.05070619	GSE40231	0.009998	0.012222
GSE17800	0.009999983	0.06984682	GSE48060	0.01	0.022985
GSE1869	0.009998259	0.02478318	GSE49925	0.01	0.019608
GSE2656	0.009997529	0.01210045	GSE5406	0.01	0.02285
GSE28454	0.009999107	0.03521767	GSE57338	0.009998	0.022185
GSE29111	0.009999147	0.08085013	GSE59867	0.009999	0.014418
GSE33463	0.009997801	0.0136186	GSE60993	0.009996	0.07749
GSE34198	0.009990152	0.01001249	GSE62646	0.01	0.023343
GSE34457	0.009998558	0.01073682	GSE66360	0.01	0.017437
GSE3586	0.009998676	0.1658121			

Supplementary Table S2

Disease	Disease name	GSE number	Platform	Samples	Number of edges	Number of nodes
**CAD**	Coronary artery disease	GSE40231	GPL570	278	236986	6120
		GSE12288	GPL96	222	25491	4053
**HCM**	Hypertrophic cardiomyopathy	GSE36961	GPL15389	145	50302	5432
**HF**	Heart failure	GSE1145	GPL570	90	52279	5681
		GSE2656	GPL2041	49	17940	1192
		GSE5406	GPL96	210	57256	4474
		GSE57338	GPL11532	313	95542	5675
**DCM**	Dilated cardiomyopathy	GSE3586	GPL3050	28	8761	3061
		GSE17800	GPL570	48	40547	5061
**MI**	Myocardial infarction	GSE48060	GPL570	52	101896	5832
		GSE60993	GPL6884	33	32806	4892
		GSE62646	GPL6244	98	91534	5595
		GSE66360	GPL570	99	134936	5991
		GSE28454	GPL6883	76	27271	3780
		GSE29111	GPL570	52	40189	5173
		GSE34198	GPL6102	97	356446	6033
		GSE59867	GPL6244	436	136969	5755
		GSE49925	GPL10558	338	52116	4115
**PAH**	Pulmonary hypertension	GSE33463	GPL6947	140	236397	6026
**ICM**	Ischemic cardiomyopathy	GSE1869	GPL96	37	57922	4330
**CHD**	Congenital Heart Disease	GSE34457	GPL6102	43	332679	6033

Supplementary Table S3

pathwayId	pathwayName	annMoleculeRatio	annBgRatio	pvalue
path:04110_8	Cell cycle	5/688	28/21796	0.001661
path:04920_4	Adipocytokine signaling pathway	4/688	18/21796	0.002115
path:04010_18	MAPK signaling pathway	5/688	30/21796	0.002286
path:00600_2	Sphingolipid metabolism	5/688	31/21796	0.002656
path:04720_4	Long-term potentiation	7/688	60/21796	0.00274
path:04060_54	Cytokine-cytokine receptor interaction	2/688	3/21796	0.002922
path:04140_2	Regulation of autophagy	2/688	3/21796	0.002922
path:04920_7	Adipocytokine signaling pathway	5/688	32/21796	0.003067
path:04062_4	Chemokine signaling pathway	5/688	33/21796	0.003522
path:04010_12	MAPK signaling pathway	5/688	34/21796	0.004024
path:04010_13	MAPK signaling pathway	5/688	34/21796	0.004024

**path:04920_3**	**Adipocytokine signaling pathway**	**5/688**	**34/21796**	**0.004024**
**path:04150_2**	**mTOR signaling pathway**	**5/688**	**35/21796**	**0.004575**
**path:04141_21**	**Protein processing in endoplasmic reticulum**	**4/688**	**22/21796**	**0.004575**
**path:04622_1**	**RIG-I-like receptor signaling pathway**	**4/688**	**23/21796**	**0.005402**
**path:04622_6**	**RIG-I-like receptor signaling pathway**	**3/688**	**12/21796**	**0.005566**
**path:00970_1**	**Aminoacyl-tRNA biosynthesis**	**2/688**	**4/21796**	**0.005722**
**path:04010_1**	**MAPK signaling pathway**	**5/688**	**37/21796**	**0.005834**
**path:04010_4**	**MAPK signaling pathway**	**5/688**	**38/21796**	**0.006547**
**path:04720_5**	**Long-term potentiation**	**6/688**	**54/21796**	**0.006905**
**path:04270_6**	**Vascular smooth muscle contraction**	**3/688**	**13/21796**	**0.007068**
**path:04620_5**	**Toll-like receptor signaling pathway**	**3/688**	**13/21796**	**0.007068**
**path:05145_12**	**Toxoplasmosis**	**3/688**	**13/21796**	**0.007068**
**path:04350_6**	**TGF-beta signaling pathway**	**4/688**	**25/21796**	**0.007343**
**path:04720_3**	**Long-term potentiation**	**6/688**	**56/21796**	**0.008234**
**path:04350_1**	**TGF-beta signaling pathway**	**4/688**	**26/21796**	**0.008465**
**path:04670_18**	**Leukocyte transendothelial migration**	**4/688**	**26/21796**	**0.008465**
**path:04670_20**	**Leukocyte transendothelial migration**	**4/688**	**26/21796**	**0.008465**
**path:04141_16**	**Protein processing in endoplasmic reticulum**	**3/688**	**14/21796**	**0.008787**
**path:04141_23**	**Protein processing in endoplasmic reticulum**	**3/688**	**14/21796**	**0.008787**
**path:05215_5**	**Prostate cancer**	**5/688**	**41/21796**	**0.009048**
**path:05210_4**	**Colorectal cancer**	**2/688**	**5/21796**	**0.009338**

Supplementary Table S4

**pathwayId**	**pathwayName**	**annMoleculeRatio**	**annBgRatio**	**pvalue**
**path:04350_8**	**TGF-beta signaling pathway**	**6/42**	**27/26232**	**3.35E-12**
**path:04110_16**	**Cell cycle**	**6/42**	**47/26232**	**1.19E-10**
**path:04350_1**	**TGF-beta signaling pathway**	**5/42**	**25/26232**	**4.27E-10**
**path:04110_11**	**Cell cycle**	**5/42**	**46/26232**	**1.07E-08**
**path:04110_15**	**Cell cycle**	**5/42**	**47/26232**	**1.20E-08**
**path:04110_12**	**Cell cycle**	**5/42**	**51/26232**	**1.83E-08**
**path:04110_3**	**Cell cycle**	**5/42**	**55/26232**	**2.70E-08**
**path:04350_6**	**TGF-beta signaling pathway**	**4/42**	**25/26232**	**7.01E-08**
**path:04010_3**	**MAPK signaling pathway**	**5/42**	**69/26232**	**8.57E-08**
**path:04114_8**	**Oocyte meiosis**	**4/42**	**28/26232**	**1.13E-07**
**path:04110_14**	**Cell cycle**	**4/42**	**30/26232**	**1.51E-07**
**path:04722_5**	**Neurotrophin signaling pathway**	**4/42**	**31/26232**	**1.73E-07**
**path:04914_4**	**Progesterone-mediated oocyte maturation**	**3/42**	**10/26232**	**4.54E-07**
**path:04722_4**	**Neurotrophin signaling pathway**	**4/42**	**42/26232**	**6.08E-07**
**path:04010_24**	**MAPK signaling pathway**	**4/42**	**47/26232**	**9.63E-07**
**path:04144_9**	**Endocytosis**	**3/42**	**15/26232**	**1.71E-06**
**path:04350_4**	**TGF-beta signaling pathway**	**3/42**	**15/26232**	**1.71E-06**

**path:04722_8**	**Neurotrophin signaling pathway**	**3/42**	**16/26232**	**2.11E-06**
**path:04010_22**	**MAPK signaling pathway**	**4/42**	**63/26232**	**3.16E-06**
**path:04010_23**	**MAPK signaling pathway**	**4/42**	**67/26232**	**4.04E-06**
**path:04350_5**	**TGF-beta signaling pathway**	**3/42**	**21/26232**	**4.97E-06**
**path:04350_7**	**TGF-beta signaling pathway**	**3/42**	**22/26232**	**5.75E-06**
**path:00512_1**	**Mucin type O-Glycan biosynthesis**	**3/42**	**26/26232**	**9.67E-06**
**path:04144_2**	**Endocytosis**	**3/42**	**37/26232**	**2.85E-05**
**path:04110_1**	**Cell cycle**	**3/42**	**40/26232**	**3.62E-05**
**path:04010_11**	**MAPK signaling pathway**	**3/42**	**56/26232**	**9.97E-05**
**path:04010_10**	**MAPK signaling pathway**	**3/42**	**57/26232**	**0.000105**
**path:04914_2**	**Progesterone-mediated oocyte maturation**	**2/42**	**10/26232**	**0.000112**
**path:04720_2**	**Long-term potentiation**	**3/42**	**60/26232**	**0.000123**
**path:04720_4**	**Long-term potentiation**	**3/42**	**60/26232**	**0.000123**
**path:05134_9**	**Legionellosis**	**2/42**	**11/26232**	**0.000136**
**path:04722_3**	**Neurotrophin signaling pathway**	**3/42**	**69/26232**	**0.000186**
**path:04914_7**	**Progesterone-mediated oocyte maturation**	**2/42**	**13/26232**	**0.000193**
**path:05145_11**	**Toxoplasmosis**	**2/42**	**13/26232**	**0.000193**
**path:04114_1**	**Oocyte meiosis**	**2/42**	**14/26232**	**0.000225**
**path:04114_2**	**Oocyte meiosis**	**2/42**	**14/26232**	**0.000225**
**path:04141_16**	**Protein processing in endoplasmic reticulum**	**2/42**	**14/26232**	**0.000225**
**path:04144_10**	**Endocytosis**	**2/42**	**14/26232**	**0.000225**
**path:04110_10**	**Cell cycle**	**2/42**	**16/26232**	**0.000296**
**path:04012_12**	**ErbB signaling pathway**	**2/42**	**17/26232**	**0.000335**
**path:04064_4**	**NF-kappa B signaling pathway**	**2/42**	**18/26232**	**0.000377**
**path:04110_4**	**Cell cycle**	**2/42**	**19/26232**	**0.000421**
**path:05164_15**	**Influenza A**	**2/42**	**19/26232**	**0.000421**
**path:04914_6**	**Progesterone-mediated oocyte maturation**	**2/42**	**21/26232**	**0.000515**
**path:04010_17**	**MAPK signaling pathway**	**2/42**	**22/26232**	**0.000566**
**path:04141_21**	**Protein processing in endoplasmic reticulum**	**2/42**	**22/26232**	**0.000566**
**path:04010_19**	**MAPK signaling pathway**	**2/42**	**23/26232**	**0.00062**
**path:04150_5**	**mTOR signaling pathway**	**2/42**	**23/26232**	**0.00062**
**path:04150_3**	**mTOR signaling pathway**	**2/42**	**28/26232**	**0.000921**
**path:04210_7**	**Apoptosis**	**2/42**	**28/26232**	**0.000921**
**path:04150_8**	**mTOR signaling pathway**	**2/42**	**30/26232**	**0.001058**
**path:04012_3**	**ErbB signaling pathway**	**2/42**	**32/26232**	**0.001204**
**path:04012_4**	**ErbB signaling pathway**	**2/42**	**32/26232**	**0.001204**
**path:04010_20**	**MAPK signaling pathway**	**2/42**	**33/26232**	**0.00128**
**path:04010_1**	**MAPK signaling pathway**	**2/42**	**37/26232**	**0.001609**

**path:04910_2**	**Insulin signaling pathway**	**2/42**	**39/26232**	**0.001786**
**path:04713_11**	**Circadian entrainment**	**2/42**	**42/26232**	**0.002069**
**path:04012_1**	**ErbB signaling pathway**	**2/42**	**43/26232**	**0.002168**
**path:04010_9**	**MAPK signaling pathway**	**2/42**	**44/26232**	**0.002269**
**path:04012_11**	**ErbB signaling pathway**	**2/42**	**45/26232**	**0.002372**
**path:04012_7**	**ErbB signaling pathway**	**2/42**	**47/26232**	**0.002585**
**path:04713_13**	**Circadian entrainment**	**2/42**	**47/26232**	**0.002585**
**path:04713_14**	**Circadian entrainment**	**2/42**	**49/26232**	**0.002806**
**path:04713_3**	**Circadian entrainment**	**2/42**	**50/26232**	**0.00292**
**path:04672_12**	**Intestinal immune network for IgA production**	**1/42**	**2/26232**	**0.0032**
**path:04380_3**	**Osteoclast differentiation**	**2/42**	**53/26232**	**0.003275**
**path:04010_16**	**MAPK signaling pathway**	**2/42**	**54/26232**	**0.003397**
**path:04713_9**	**Circadian entrainment**	**2/42**	**55/26232**	**0.003522**
**path:04010_15**	**MAPK signaling pathway**	**2/42**	**59/26232**	**0.004041**
**path:04010_2**	**MAPK signaling pathway**	**2/42**	**61/26232**	**0.004314**
**path:04510_12**	**Focal adhesion**	**2/42**	**63/26232**	**0.004594**
**path:04910_7**	**Insulin signaling pathway**	**2/42**	**64/26232**	**0.004738**
**path:04150_4**	**mTOR signaling pathway**	**1/42**	**3/26232**	**0.004796**
**path:04151_5**	**PI3K-Akt signaling pathway**	**2/42**	**71/26232**	**0.005799**
**path:04010_14**	**MAPK signaling pathway**	**1/42**	**4/26232**	**0.006389**
**path:04380_14**	**Osteoclast differentiation**	**1/42**	**4/26232**	**0.006389**
**path:05142_7**	**Chagas disease (American trypanosomiasis)**	**1/42**	**4/26232**	**0.006389**
**path:05323_5**	**Rheumatoid arthritis**	**1/42**	**4/26232**	**0.006389**
**path:04510_5**	**Focal adhesion**	**2/42**	**75/26232**	**0.006449**
**path:04060_23**	**Cytokine-cytokine receptor interaction**	**1/42**	**5/26232**	**0.007981**
**path:04145_3**	**Phagosome**	**1/42**	**5/26232**	**0.007981**
**path:04612_6**	**Antigen processing and presentation**	**1/42**	**5/26232**	**0.007981**
**path:04726_4**	**Serotonergic synapse**	**1/42**	**5/26232**	**0.007981**
**path:05210_4**	**Colorectal cancer**	**1/42**	**5/26232**	**0.007981**
**path:05220_5**	**Chronic myeloid leukemia**	**1/42**	**5/26232**	**0.007981**
**path:04320_6**	**Dorso-ventral axis formation**	**1/42**	**6/26232**	**0.009569**
**path:04350_2**	**TGF-beta signaling pathway**	**1/42**	**6/26232**	**0.009569**
**path:04350_3**	**TGF-beta signaling pathway**	**1/42**	**6/26232**	**0.009569**
**path:05161_25**	**Hepatitis B**	**1/42**	**6/26232**	**0.009569**
**path:05169_5**	**Epstein-Barr virus infection**	**1/42**	**6/26232**	**0.009569**

Supplementary Table S5

**pathwayId**	**pathwayName**	**annMoleculeRatio**	**annBgRatio**	**pvalue**
**path:04612_2**	**Antigen processing and presentation**	**1/38**	**8/26232**	**0.011532**
**path:04141_8**	**Protein processing in endoplasmic reticulum**	**1/38**	**12/26232**	**0.017249**

**path:04722_8**	**Neurotrophin signaling pathway**	**1/38**	**16/26232**	**0.022934**
**path:04916_6**	**Melanogenesis**	**1/38**	**18/26232**	**0.025765**
**path:04962_2**	**Vasopressin-regulated water reabsorption**	**1/38**	**20/26232**	**0.028587**
**path:05215_4**	**Prostate cancer**	**1/38**	**21/26232**	**0.029995**
**path:04722_5**	**Neurotrophin signaling pathway**	**1/38**	**31/26232**	**0.043969**
**path:04630_1**	**Jak-STAT signaling pathway**	**1/38**	**38/26232**	**0.053634**
**path:05215_5**	**Prostate cancer**	**1/38**	**41/26232**	**0.057747**
**path:04010_9**	**MAPK signaling pathway**	**1/38**	**43/26232**	**0.060479**
**path:04722_4**	**Neurotrophin signaling pathway**	**1/38**	**43/26232**	**0.060479**
**path:05215_10**	**Prostate cancer**	**1/38**	**44/26232**	**0.061843**
**path:04010_11**	**MAPK signaling pathway**	**1/38**	**55/26232**	**0.076712**
**path:04010_10**	**MAPK signaling pathway**	**1/38**	**56/26232**	**0.078052**
**path:04010_22**	**MAPK signaling pathway**	**1/38**	**63/26232**	**0.087381**
**path:04630_2**	**Jak-STAT signaling pathway**	**1/38**	**87/26232**	**0.118674**

Supplementary Table legends:

Supplementary Table S1: Comparison and analysis of p-values and FDR that calculated from Pearson correlation coefficients. We calculated p-values and corresponding FDR values for candidate ceRNA interactions in each gene expression profile. In each gene expression profile, we listed the maximum p-value and its adjusted FDR value in the table.

Supplementary Table S2: The summary of data in this study. In our study, we downloaded 21 gene expression profiles encompassed 8 CVDs. We listed the disease name, GSE number, platform, samples, number of edges and number of nodes in each network.

Supplementary Table S3: Subpathway enrichment analysis of ceRNAs in common core ceRNA network. In total, 32 subpathways were enriched by mapping the ceRNAs in common core ceRNA network into enrichment program.

Supplementary Table S4: Subpathway enrichment analysis of common hub CDC40. Some CVD related pathways were enriched, indicating that CDC40 and its interacted partners exerted similar functions in multi-CVDs.

Supplementary Table S5: Subpathway enrichment analysis of differential hub BCL11A. Different subpathways were enriched in different disease indicated that the potential different roles of BCL11A in CVDs.

Supplementary Table S6: List of common ceRNA modules in our study. In total, we identified 131 common modules in this study.
